# Nano-scale insights regarding coke formation in zeolite SSZ-13 subject to the methanol-to-hydrocarbons reaction†

**DOI:** 10.1039/d1cy01938d

**Published:** 2022-01-08

**Authors:** S. H. van Vreeswijk, M. Monai, R. Oord, J. E. Schmidt, E. T. C. Vogt, J. D. Poplawsky, B. M. Weckhuysen

**Affiliations:** Inorganic Chemistry and Catalysis Group, Debye Institute for Nanomaterials Science, Utrecht University Universiteitsweg 99 3584 CG Utrecht The Netherlands e.t.c.vogt@uu.nl b.m.weckhuysen@uu.nl; Center for Nanophase Materials Sciences, Oak Ridge National Laboratory Oak Ridge TN 37831 USA poplawskyjd@ornl.gov

## Abstract

The methanol-to-hydrocarbons (MTH) process, commonly catalyzed by zeolites, is of great commercial interest and therefore widely studied both in industry and academia. However, zeolite-based catalyst materials are notoriously hard to study at the nano-scale. Atom probe tomography (APT) is uniquely positioned among the suite of characterization techniques, as it can provide 3D chemical information with sub-nm resolution. In this work, we have used APT to study the nano-scale coking behavior of zeolite SSZ-13 and its relation to bulk coke formation on the macro-/micro-scale studied with *operando* and *in situ* UV-vis spectroscopy and microscopy. Radial distribution function analysis (RDF) of the APT data revealed short carbon–carbon length scale affinities, consistent with the formation of larger aromatic molecules (coke species). Using nearest neighbor distribution (NND) analysis, an increase in the homogeneity of carbon was found with increasing time-on-stream. However, carbon clusters could not be isolated due to spatial noise and limited clustering. Therefore, it was found that the coke formation in zeolite SSZ-13 (CHA) is reasonably homogeneous on the nano-scale, and is rather similar to the silicoaluminophosphate analogue SAPO-34 (CHA) but different in nano-scale coking behavior compared to previously studied zeolite ZSM-5 (MFI).

## Abstract 2

A correlation between the micro- and nano-scale coking behavior of SSZ-13 was discovered with *in situ*/*operando* spectroscopy and atom probe tomography (APT), which allows for spatial reconstruction and analysis of relations between framework elements and carbon atoms.

## Graphical abstract

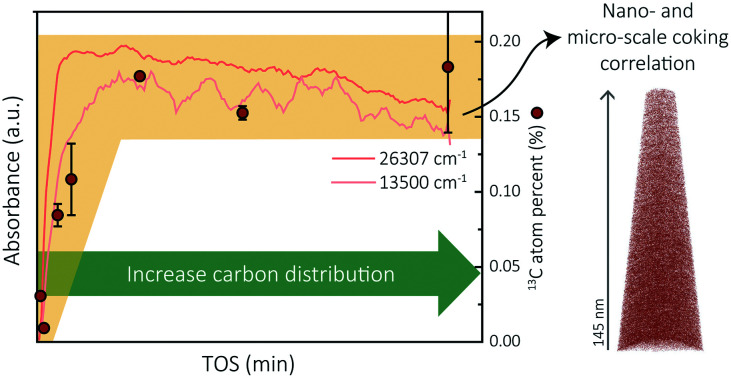

## Introduction

In the methanol-to-hydrocarbons (MTH) process, methanol is efficiently converted into hydrocarbons over zeolite-based catalyst materials. The nature of the hydrocarbons formed in the MTH process is determined by a variety of factors, such as the reaction conditions and the catalyst properties.^1,2^ The MTH process is of great commercial and industrial interest as methanol can be produced from almost any carbon containing source, including renewable sources, such as biomass, municipal waste and even directly from CO_2_.^3–5^ Therefore, this catalytic process provides an alternative to fossil-based production routes for making products such as plastics and transportation fuels.^6–9^ The MTH reaction is catalyzed by a wide variety of zeolites and zeotype materials. The properties of the porous structure of these materials are of major importance for the catalytic performance as they control activity, selectivity as well as stability.^2,10^ Small-pore zeotype materials, such as SSZ-13 and SAPO-34 (both of CHA structure), are, for instance, highly selective towards light olefins and the related process is called the methanol-to-olefins (MTO) reaction. In contrast, medium-pore zeolites, such as ZSM-5 (MFI), allow larger hydrocarbons to diffuse out of their micropores, which opens the possibility to perform the methanol-to-aromatics (MTA) reaction.^11,12^ Besides these structural properties, the acidic properties originating from the chemical composition of the zeolite material are also very important.^2,13^ Indeed, the acidic properties can be altered by changing the framework elements and their ratios (*e.g.*, P can be replaced by Si leading to a framework containing Al, Si and P) and by changing the specific location of these elements in the zeolite framework.^14–17^ This also means that zeolite-based materials with the same framework structures could have very different acidic sites. For instance, SAPO-34 and SSZ-13, although possessing the same framework structure (*i.e.*, CHA), have different framework elements and therefore the strength of the acid sites are different (*i.e.*, they are stronger in SSZ-13 than in SAPO-34).^18–20^ A key component in the MTH process is believed to be the formation of the so-called hydrocarbon pool (HCP).^18,21^ The precise molecular composition of the HCP is mainly depending on the reaction conditions and the properties of the zeolite material, but does consist of olefinic and aromatic molecules trapped inside the zeolite channels and cavities, and strongly influences the final reaction product selectivity.^19,22–25^ However, if these aromatic intermediate compounds grow too large, they can also deactivate the catalyst.^18^ The formation of undesired coke molecules is well studied from the bulk to the micro-scale. However, unraveling the coking behavior with sub-nm resolution remains a challenge. Previous research,^26–28^ has proven that atom probe tomography (APT) is a unique and suitable technique to investigate zeolite-based materials as it can provide information about the chemical composition with sub-nm resolution in 3D. APT is also not hindered by the limited *z*-contrast (atom number contrast) difference of the framework elements of the zeolite.^29^ To the best of our knowledge, the APT technique is currently unique in its capability to determine 3D nanometer scale relationships between light elements in these materials.^30^

In this work, new nano-scale relationships are established for zeolite SSZ-13 with APT, which are compared with those previously uncovered for zeolite ZSM-5 and molecular sieve SAPO-34 (ref. 30, 27 and 29) with the general aim to better understand the influence of the framework structure and chemical composition on coke formation. Additionally, we have studied coke deposition on the nano-scale as a function of time-on-stream (TOS) to relate the bulk coking behavior as a function of TOS. The bulk and single crystal coking behavior has been studied with *operando* and *in situ* UV-vis spectroscopy and microscopy.

## Experimental

### Catalyst preparation

Zeolite H-SSZ-13 was synthesized as described in Oord *et al.*^31^ A structure directing agent (SDA, *N*,*N*,*N*-trimethyl-1-adamantammonium, 20 wt%, Sachem, pure) was added to tetra-ethyl orthosilicate (TEOS, Aldrich, >99%) and aluminum isopropoxide (Acros Organics, 98%+). The resulting mixture was aged at RT for about 4 days until ±100 mg had evaporated. A 51% HF solution (Acros organics, 48–51%) was added to the materials and it was stirred up to the point that a homogeneous mixture is obtained as a gel-like substance. The resulting gel was then transferred into three Teflon-lined autoclaves, in equal portions. The autoclaves were sealed and put in a static oven at 150 °C for 6 days. After the synthesis was finished, the resulting solid was washed thoroughly (∼8 L) with demineralized water. The resulting solid was a white powder. The SDA was burned away during calcination. The calcination was performed in a static air oven at 580 °C for 5 h using a heating rate of 1 °C min^−1^ to reach the maximum temperature of calcination. The crystallinity and phase purity of the obtained material was evaluated with X-ray diffraction (XRD) performed with a Bruker D2 Phaser diffractometer. The instrument makes use of a fixed slit and Co-K_α_ X-ray tube (*λ* = 1.79026 Å). The XRD patterns are compared with database patterned for the catalyst obtained from Powder Diffraction File™ (PDF-4).

### ^13^C coking experiments

The SSZ-13 crystals were coked for APT studies in a Linkam cell starting with a pretreatment step at 150 °C (5 °C min^−1^) under a N_2_ flow (5 mL min^−1^) and kept there for 30 min followed by a heating step to 450 °C under a N_2_ flow (5 mL min^−1^). MTH coking reaction was performed at 450 °C flowing a N_2_ flow of 5 mL min^−1^ through a saturator filled with ^13^C-labelled methanol (Sigma Aldrich, 99 at%). ^13^C-Labelled methanol was used to avoid interference from adventitious ^12^C during APT analysis. Possible isotopic effects on coke formation kinetics are limited to 8% by the relative mass of the carbon isotopes. The MTH reaction was performed for 0.5, 1, 3, 5, 15, 30, 60 and 120 min (for the 120 min experiments a flow of 20 mL min^−1^ was used).

### *In situ* UV-vis microscopy

**Fig. 1:**
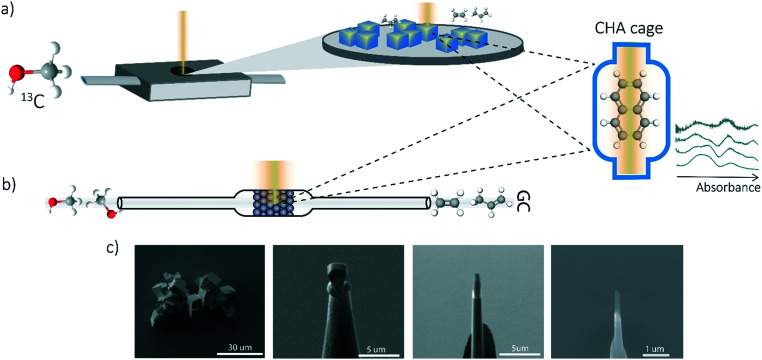
Schematic representation of some experimental methods. a) Single-crystal analysis with *in situ* UV-vis microscopy using a Linkam cell and a UV-vis microscope, b) *operando* UV-vis spectroscopy to study activity–coking relations on the bulk-scale, c) SEM images of the needle preparation for the APT experiments.

A similar procedure has been performed as the coking experiments for the APT samples only normal ^12^C methanol (Acros, HPLC grade, 99.99% pure) was used to perform this experiment. The UV-vis DRS measurements were performed with a CRAIC 20/30 PV UV-vis-NIR micro-spectrophotometer using a 36× objective. A 75 W xenon lamp was used for illumination. The absorbance measured at different wavelengths have been analyzed and compared to the bulk *operando* UV-vis spectroscopy to obtain single-crystal coking information over time. A schematic representation of the *in situ* UV-vis microscopy experiments and the coking procedure is depicted in Fig. 1a.

### *Operando* UV-vis diffuse reflectance spectroscopy

UV-vis diffuse reflectance spectroscopy (DRS) of the zeolite material was combined with on-line activity measurements with a gas chromatograph (GC). For this purpose, a quartz, rectangular fixed bed reactor was used and the details of this setup can be found in previous articles from our group.^11,19,32,33^ Methanol saturation was generated using a He flow through a saturator containing methanol at a specific temperature (*i.e.*, 21 °C). Before starting the actual MTH reaction, the sample was first calcined under a 10 ml min^−1^ O_2_ flow at 550 °C (with a heating rate 5 °C min^−1^). Next, the sample was cooled down to the reaction temperature of 450 °C under a 35 ml min^−1^ He flow. A weight-hourly space velocity (WHSV) of 1.3 h^−1^ was used to obtain information about the activity of the zeolites (*e.g.*, 60 mg zeolite, *ca.* 14% methanol saturation and 6.5 ml min^−1^ He flow for 300 min). UV-vis DRS was performed with an AvaSpec2048L spectrometer connected to a UV-vis probe. With an on-line Interscience Compact GC, RTX-wax and RTX-1 column (in series connected to FID detector) and Rtx-1, Rt-TCEP and Al_2_O_3_/Na_2_SO_4_ (in series connected to FID detector), the reactants and reaction products were analyzed. GC data analysis is done by calculating the methanol conversion and the product yield. A schematic representation of the *operando* UV-vis experiment is depicted in Fig. 1b.

### Atom probe tomography (APT)

Measurements were performed at Oak Ridge National Laboratory (ORNL). The needles used for the APT analysis were prepared as described by Schmidt *et al.* using an established focused ion beam-scanning electron microscopy (FIB-SEM) specimen preparation technique.^29^ A Si micro-tip, purchased from CAMECA, was used to perform this FIB-milling technique. SEM pictures of the needle preparation procedure are shown in Fig. 1c additionally with a schematic representation in the ESI† section 2. At least one needle of each time-on-stream point was successfully prepared. When more needles of the same samples are prepared, the statistical information is increased and error bars can be included in the results. The measurements were performed using a LEAP 4000XHR local electrode atom probe in laser mode using a 200 pJ laser pulse energy, a 40 K base temperature, and a 0.5–2.0 detection rate. All APT analysis has been performed with CAMECA's integrated visualization and analysis software (IVAS).

The theory about the different data analysis techniques has been extensively explained in various previous publications.^29,30^ Both the nearest neighbor distribution (NND) analysis and radial distribution function analysis provide information about the distribution of elements in the measured needles and heterogeneities can be found. In NND analysis, the measured pair distances between certain elements is compared to the randomized pair distance (distribution if all ions of that element would have been homogeneously distributed through the needle). When the measured pair-distances of a certain element are shorter than the randomized pair distances, this indicates a heterogeneous distribution. RDF is comparing the short length-scale concentrations (local concentrations) to the longer length-scale (bulk) concentrations. In this way, short length-scale affinities between elements and heterogeneously distributed elements can be found. More detailed explanation of NND and RDF analysis can be found in the ESI† in section S3.3 and S3.5, respectively. To quantify the difference between the measured NND and randomized NNDs, the Pearson coefficient between these NNDs has been calculated for each sample.^29,30,34,35^ A *χ*^2^ statistical test can be applied as a measure to quantify the difference between the measured and the randomized normal distribution. In this case, *χ*^2^ is identified as *X*^2^, when taking 5 counts as a minimum condition. The *X*^2^ can be calculated as:^35,36^1
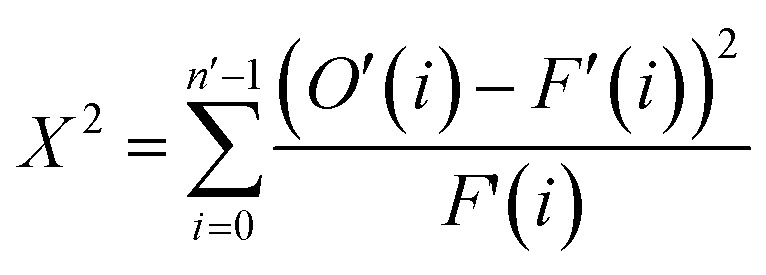
Here, the *O*′ are the experimental observed counts for each distance, *F*′ the randomized counts for each distance and *n*′ the total amount of measured distances (with at least 5 counts). However, the use of only the *X*^2^ statistical test to identify and quantify the deviation of the randomized/calculated data and the measured data is not sufficient as it is very sensitive to large and deviating data set numbers. Therefore, the Pearson coefficient (*μ*) is used in this study as it is normalizing the *X*^2^ statistical test to the sample size (the total number of counts (*N*_s_)).^35^2
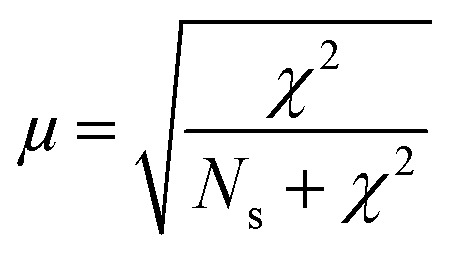
Another statistical method to quantify the deviation in the NND from a random distribution has been used as well, in which the average of two fitted Gaussian curves (experimental and calculated distributions) are compared. The fits can be found in the ESI† in section S3.4.

## Results and discussion

### Bulk coking behavior studied with *in situ* and *operando* UV-vis spectroscopy

*Operando* UV-vis and *in situ* UV-vis microscopy experiments have been performed to prove that the bulk coking behavior (studied with *operando* UV-vis spectroscopy) can be compared to the single crystal coking behavior (studied with *in situ* UV-vis microscopy) even though the reaction time-scale and reactor set-ups are completely different. Additionally, these experiments show that the coking behavior is linked to the methanol conversion and thereby the deactivation of SSZ-13 zeolite.

**Fig. 2:**
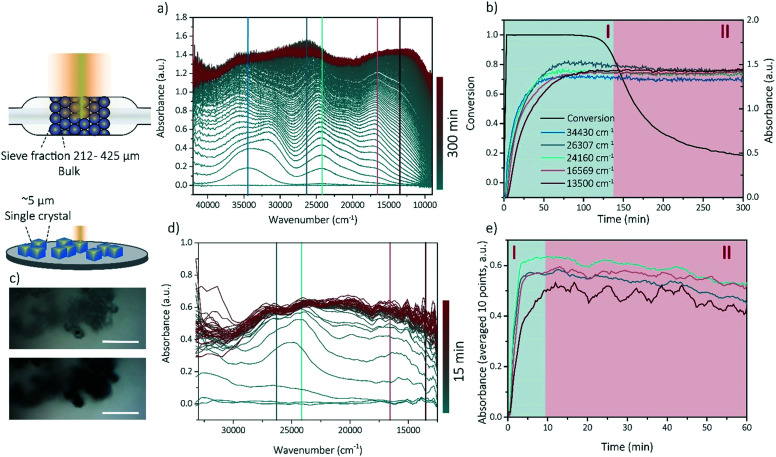
*Operando* UV-vis spectroscopy and *in situ* UV-vis microscopy results. a) *Operando* UV-vis results with reaction time (from blue to red = 300 min), wavenumbers specific to clear bands are indicated with lines and further UV-vis analysis will be performed at these wavenumbers. b) Evolution of the intensity of the absorbance at specific wavelengths over time in the *operando* UV-vis spectroscopy experiments and its corresponding methanol conversion profile with time-on-stream. c) Optical microphotographs taken after 20 s and 60 min time-on-stream (TOS). Scale bar represents 50 μm. d) *In situ* UV-vis spectra obtained with the *in situ* microscopy experiment on single SSZ-13 crystals. Spectra are binned to 4 nm and a moving average of 5 points is used to get rid of undesired noise. e) Evolution of the intensity of the absorbance at the specific wavelengths.

*Operando* UV-vis spectroscopy experiments can provide information about activity-intermediate relations within zeolite SSZ-13. The *operando* UV-vis spectra are depicted within Fig. 2a, with the wavenumbers clearly corresponding to rising UV-vis bands indicated by colored lines. Further analysis of the UV-vis spectra is performed by using these specific wavenumbers and determine the evolution of the intensity of the absorbance over time (Fig. 2b) without deconvoluting the spectra as coke formation can make this unnecessarily difficult. We therefore describe the intensity changes at certain wavelengths as bands even though they are not real Gaussian band intensities. Because we use the UV-vis data to obtain information about activating and deactivating trends, and no precise qualification nor quantification is necessary, we are on the opinion that this approach is sufficient. Bands contributing to an increase in the UV-vis spectra at smaller wavelengths are normally attributed to the formation of aromatic intermediates or hydrocarbon pool (HCP) species (40 000–23 000 cm^−1^), while lower wavenumbers normally correspond to the formation of higher alkylated and conjugated aromatic compounds (coke precursors, 23 000–9500 cm^−1^). For instance, the increase at 34 430 cm^−1^ can correspond to the formation of less alkylated benzenes and/or charged mono-enyl/cyclopentenyl species, while the bands around 26 307 cm^−1^ and 24 160 cm^−1^ could indicate the formation of charged poly-alkylated benzenes species and (charged) poly-alkylated naphthalenes, respectively.^11,19,33,37^ Bands at wavenumbers 16 569 and 13 500 cm^−1^ both correspond to the formation of charged and neutral polyaromatic species.^11,19,33,37,38^ All bands in the UV-vis spectra increase in intensity with reaction time and are stabilizing after the conversion is decreasing (deactivation), with the bands corresponding to poly-aromatic compounds which is intuitive with the contribution to the deactivation of these components. This means that there is a strong correlation between the catalyst lifetime and performance and the development of the coke molecules. We are therefore using the *operando* UV-vis spectra to develop a method to identify the phase of the reaction by their UV-vis patterns. We were able to distinguish between two reaction stages: the activating period (stage I), in which the conversion is above 80% and the aromatic intermediates and coke precursors are increasing and the deactivating period (stage II) in which the conversion is decreasing below 80% and the formation of aromatic intermediates and polyaromatic compounds is stabilizing.

As all samples for the APT experiments are prepared (coked) *via* a reaction performed in a Linkam cell, the reaction stage can be determined by performing single-crystal UV-vis spectroscopy/microscopy. The single-crystals are darkening with increasing reaction time. Some optical microphotographs are shown in Fig. 2c and in the ESI† in section 1. Visually, from 5 min time-on-stream, no differences can be observed. A strong correlation was found between the macro (-bulk) and the micro-scale (single crystal) coking behavior. Bands arise at similar positions in the UV-vis spectra of both the *in situ* (single crystal) microscopy experiments and *operando* (bulk) spectroscopy, which indicates that the same reaction intermediates and coke precursors are formed within the two different set-ups and the reaction mechanisms are comparable on these two different length scales (Fig. 2a and d) Therefore, the bulk coking behavior can be compared with the coking behavior on single-crystal level. Consequently, with this method, the two reaction stages (I and II) could be identified on the single-crystal level as well (Fig. 2e) and time-on-stream points could be carefully chosen for nano-scale coking behavior analysis with APT.

### Nano-scale coking studied with atom probe tomography

To gain more insights into the observed coking behavior of zeolite SSZ-13, we have performed APT analysis on the spent catalysts. We chose four time-on-stream (TOS) points from stage (I), for which the development of the coke (and thereby active and deactivating compounds) was ongoing: 0.5, 1, 3 and 5 min. We also chose four TOS points from stage (II), at which coke formation was already at plateau: 15, 30, 60 and 120 min.

**Fig. 3:**
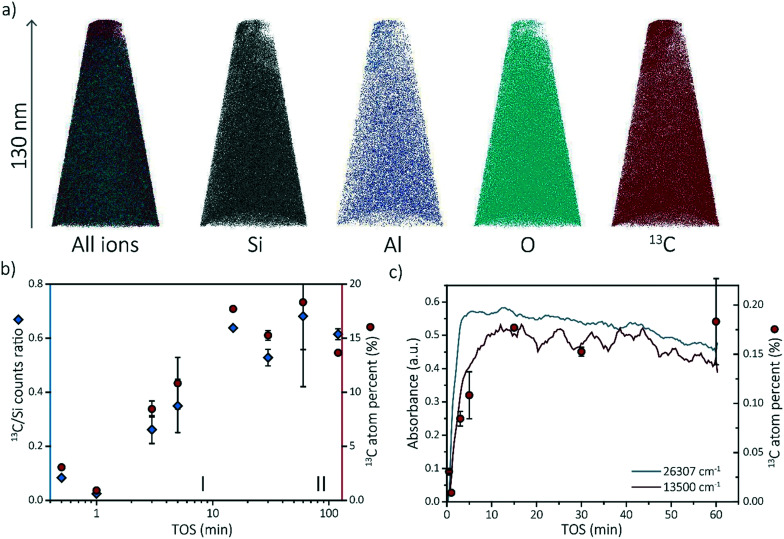
a) Reconstructed needle (ion map of framework and coke molecule ions) of the 3 min coked H-SSZ-13 sample (2). b) The average ^13^C/Si counts ratio and ^13^C atomic fraction for the reconstructed needles as a function of time-on-stream (TOS) on a log-scale to amplify on the first reaction stage. c) The carbon content found with atom probe tomography compared to two different wavelength intensity evolutions of the single crystal UV-vis microscopy experiments showing similar trends. The 120 min has been left out for clarity reasons. For the 1 min and 15 min time-on-stream sample, only one needle has been reconstructed so no error bars are available.

At least one needle per sample was successfully reconstructed. The different ion maps of one of the reconstructed needles are shown in Fig. 3a. All the other reconstructed needles can be found in section S3.1 of the ESI.† The compositions of each APT dataset are displayed in Tables S1–S8 of the ESI.† Comparing the compositions of the needles, two trends are observed. First, the composition between needles of the same sample varies. For instance, the percentage of coke in the 60 min coked samples varies between 21.4% and 15.2% (resulting in a larger error bar in the ^13^C atom percent data in Fig. 3c). This is an indication that the coke is, on the bulk and microscale scale, heterogeneously distributed. Although the needles were attempted to be fabricated in the same manner by extracting them from a similar depth from the crystal surface (∼50 ± 50 nm below crystal surface), this could be due to the different location of the needles as a non-uniform distribution of coke species has been described by Gao *et al.*^39^ Second, the amount of coke found with APT increases with increasing time-on-stream. Fig. 3b depicts the ^13^C/Si counts ratio as well as the ^13^C atomic fraction found on average in the reconstructed needles with time-on-stream on a log scale to clarify the increase in reaction stage I. The ^13^C/Si ratio has been added to show that the ^13^C atomic fraction is quantitative and consistent with bulk analysis. This is done to reduce the importance of the total counts and the possible fluctuations of other atoms. For most of the samples, multiple APT datasets were acquired and the standard deviation between datasets is reported as error bars (except for 1 min and 15 min TOS points as only one needle was successfully reconstructed). The nano-scale coke composition found with APT shows great similarities with the *in situ* UV-vis spectroscopy data as both show an increase in the number of coke molecules (C concentration) from 0–15 min TOS and a relatively constant number of coke species for TOS >15 min. This coherence is highlighted in Fig. 3c. This means that there is a strong relationship between the macro-/micro-scale and the nano-scale coking behavior in zeolite SSZ-13 used in the MTH reaction. Additionally, it means that it is possible to define the “reaction stages” on the nano-scale as well, which means we can use this approach to study the nano-scale coking behavior in the activating as well as in the deactivating reaction period.

### Carbon distribution at the nano-scale

**Fig. 4:**
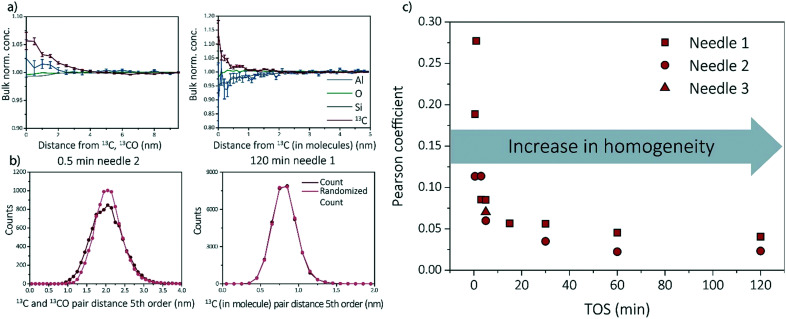
a) Radial distribution analysis (RDF) and b) fifth order nearest neighbor distribution (NND) analysis of the 0.5 and 120 min sample. c) The Pearson coefficient with time-on-stream (TOS) showing an evidenced increasing homogeneity.

Multiple data analysis techniques are available to investigate the atom distribution on the nano-scale. In this study, we focus on two commonly used techniques in APT research: the nearest neighbor distribution (NND) analysis and radial distribution analysis (RDF). Explanation and all results can be found in ESI† (section S3.3 and S3.5) as well as some additional analysis methods, such as iso-surface analysis (section S3.2 of the ESI†). For the RDFs, the bulk normalized composition is presented. Higher normalized compositions at shorter distances are indicative of clustering. All RDFs are shown in section S3.5 of the ESI† and selected RDF plots are displayed in Fig. 4a (shortest and longest TOS points). All radial distribution analysis results show that the normalized concentration of ^13^C atoms, as a function of the distance to a ^13^C atom or molecule, are higher at shorter distances, indicating carbon clustering. This is very intuitive as carbon atoms are present in the zeolite as aromatic/coke molecules and are thereby, by definition, very small clusters. Another interesting result worth noting, is the possible short length scale affinity between ^13^C and Al, which appears for multiple needles. The bulk normalized concentration of Al is higher at shorter length-scales to ^13^C in the ^13^C centered RDF for the 0.5 min coked, which indicates the nano-scale relationship between coke formation and Brønsted acid sites, as previously reported for ZSM-5 and SAPO-34.^27,29^ The same phenomenon has been observed in the RDF analysis for Al centers, in which an affinity between Al and ^13^C was also observed (ESI† section 3.5).

The experimental NND is compared to an NND with randomized ionic identities weighted with the bulk composition (randomized NND). A deviation from the randomized NND indicates that there is clustering of these atoms. This analysis can be done for first order nearest neighbors, but also for larger order nearest neighbors to reduce measurement noise. All NND's are shown in section S3.3 of the ESI† and selected fifth order distributions are shown in Fig. 4b. Absolute pair distances between samples cannot be compared as for some samples larger carbonic ionic fractions have been taking into account than for other samples. It can be visually seen that the experimental NND of ^13^C atom/molecules becomes more similar to the randomized NND with respect to TOS. This means that the carbon atoms are homogeneously distributed through the sample. In the ESI† in section 3.3, the Pearson coefficient is compared to the deviation of the curves (fitted with a Gaussian curve). In Fig. 4c, the Pearson coefficients for the NNDs are compared over time. Although the Pearson coefficient is never >0.5, which would indicate moderate correlation, it is clear that the value decreases rapidly from 0.3 to 0.05 in the first 15 min and eventually stabilizes at a value of ∼0 for longer TOS.

For some needles (up to 5 min TOS), the carbon–carbon affinity found with the RDF and the slight deviations from the random distributions found with the NND, show that the carbon is clustering. However, the clustering is weak, and becomes weaker with increasing TOS, making it impossible to extract clusters using the maximum separation method due to spatial noise and possible detection efficiency. It is intuitive that there would be clusters in the data because the coke species are molecules containing multiple C atoms bound together in a non-C containing framework. Therefore, the slightly heterogeneous C distribution for lower TOS can be interpreted as the coke molecules partially filling the zeolite pores such that they are more isolated making them resolvable in the APT data. For longer TOS (15 min+), the coke molecules are filling all or most of the pores in the zeolite structure, such that they are too close together for APT to resolve the molecules due to spatial blurring.

### Comparison study of the nano-scale coking behavior of SSZ-13 with SAPO-34 and ZSM-5

Recent work of our group has focused on “aged” ZSM-5 and SAPO-34 materials.^27,29^ Therefore, we are able to compare the observed results for SSZ-13 with the other two zeotype materials. As this study focused on the effect of reaction time, and only one reaction time for ZSM-5 and SAPO-34 was studied in previous work, two different comparison perspectives are applied: 1) the reaction stage and 2) the coke content (^13^C atomic percent in the needles). From literature we know that both SAPO-34 and ZSM-5 were coked until stage II, which indicates that 15–120 min coked SSZ-13 samples should be comparable.^27,29,40^ However, as we are looking at several studies with different parameters, another angle to look at the data is also required. The amount of carbon is not the same for all samples in this case, which can be due to several factors (*e.g.*, amount/nature of acid sites and structural differences). The carbon fraction found within ZSM-5 and SAPO-34 is between ∼0–10%, while for SSZ-13 this varies between ∼0–20% depending on the reaction time. This difference in carbon content could be attributed to the acidic differences of the materials. Therefore, also the SSZ-13 results containing a carbon content between 0–10% are compared to the ZSM-5 and SAPO-34 results (TOS 0.5–3 min) as the carbon content should be comparable.

**Fig. 5:**
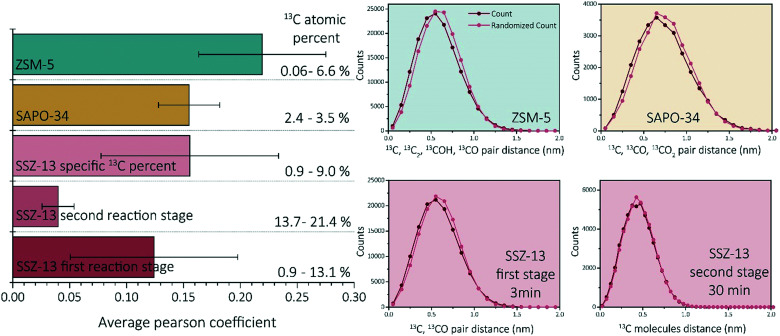
Average Pearson coefficient of ZSM-5 (data reproduced from ref. 27), SAPO-34 (data reproduced from ref. 29), SSZ-13 with a coke fraction between 0.9 and 9%, SSZ-13 in the first reaction stage (0.5–5 min) and SSZ-13 in the second reaction stage (15–120 min). Nearest neighbor distribution analysis of ZSM-5 (data reproduced from ref. 27, same needle as the C cluster isolation was performed on), SAPO-34 (data reproduced from ref. 29, same needle as the C cluster isolation was performed on), SSZ-13 3 min, and SSZ-13 30 min.

First of all, ZSM-5 contained coke depleted areas on the scale of >50 nm in the APT data, which was not observed for either SAPO-34 and SSZ-13. Second, unlike SSZ-13, for both ZSM-5 and SAPO-34, carbon clusters were identified, which were of a size consistent with known coke species.^27,29^ However, the coke clusters found in SAPO-34 were less resolvable than the C clusters in ZSM-5. We calculated the Pearson coefficient of the NNDs for the three zeolites, which are compared in Fig. 5. The comparison of the Pearson coefficients indicates that the C is more heterogeneously distributed through ZSM-5 compared to SSZ-13 (for both comparison perspectives) and SAPO-34. The increased heterogeneity in ZSM-5 can be attributed to the larger pore and C cluster sizes found in these samples because larger, more spaced-out clusters are easier to identify due to the spatial blurring of atom positions in APT data. Additionally, from literature, it is known that (large) ZSM-5 crystals are deactivated by coke agglomeration on the exterior shell of the crystals, resulting in less coke inside the pores of the zeolites, while for the chabazite structures (SSZ-13 and SAPO-34), deactivation occurs within the pores of the crystal, also resulting in lattice expansion.^17,41^ The APT data shows a more homogeneous carbon distribution for the chabazite structure than for the MFI structure, which can be explained by the infiltration of aromatic coke molecules covering all pores of the zeolite and confirms thereby the aforementioned statement. This hypothesis is enhanced by the differences observed with increasing time-on-stream for SSZ-13. In the first reaction stage, the carbon is more heterogeneously distributed, explained by smaller aromatic coke molecules decorating only a fraction of the pore free volume. Additionally, the finding of large coke depleted areas in ZSM-5 proves that the nano-scale coking behavior is largely topology dependent. Furthermore, a recent study of Wang *et al.*^42^ proposed the formation of larger cage-passing aromatic molecules as the deactivating mechanism for SAPO-34. This is also in-line with this “extensive-interior-coking principle” of the chabazite structure even on the nano-scale, resulting in less spatially resolved carbon molecules. This unique coke-growth mechanism, thereby, could also explain the clear differences between the nano-scale coking behavior of the CHA and MFI structure. The Pearson coefficient differences cannot be explained by the differences in the nature of Brønsted acidic sites as proposed in Schmidt *et al.*,^29^ as a similar near homogeneous distribution of carbon was found within this study for the aluminosilicate SSZ-13, which has the same structure, but different acidity compared to SAPO-34. When comparing the Pearson coefficients of both comparison descriptors (coke content and reaction stage), they show great similarities, and therefore SAPO-34 and SSZ-13 have a predominantly homogeneous, nano-scale coke distribution.

Indications for a short length scale affinity between carbon and the Brønsted acid sites were found for all three zeotype materials.^29^ However, more research is necessary to prove this principle in all cases and could be a very interesting subject for further zeolite – APT research as nano-scale relations and insights of carbon, but also introduced metals and aluminum helps to unravel the complex reaction mechanism. Further and better knowledge of this process can enhance catalyst design and increase *e.g.*, MTH process efficiency.

## Conclusion

The coking behavior of zeolite SSZ-13 in the MTH process was studied at the micro- and nano-scale *via* UV-vis microscopy/spectroscopy and atom probe tomography (APT). A correlation between the nano-scale carbon content, found with APT and the bulk evolution of aromatic compounds studied with *in situ* and *operando* UV-vis spectroscopy, was found, corroborating the validity of the experimental approach. Carbon–carbon short length-scale affinities were found with RDF analysis, which is intuitive, given that carbon is present in the structure as larger hydrocarbon molecules (coke). A visual inspection and *χ*^2^ analysis of the C nearest neighbor distribution analysis data shows that the C distribution becomes more homogeneous with increasing reaction time; however, the clustering was weak such that the heterogeneity was not large enough to isolate carbon clusters using the maximum separation method. The increase in homogeneity can be interpreted as the coke molecules filling all of the pores in SSZ-13, such that the coke molecules are too close together to spatially resolve the clustering of the C atoms in the molecules by APT. At lower TOS, the coke molecules are spaced further apart such that not all the pores are filled, allowing for the clustering to be resolved. Overall, the chabazite structure differs from ZSM-5 in coking behavior on the bulk scale and, as revealed by APT, the nano-scale. The difference in coking behavior of ZSM-5 compared to SSZ-13 and SAPO-34 can be explained by internal (SSZ-13 and SAPO-34)/external (ZSM-5) coke formation, spatial constrains of the framework, blurring limitations of APT and differences in Brønsted acidity.

## Author contributions

S. H. van Vreeswijk: conceptualization, methodology, investigation, formal analysis, writing – original draft, visualization. M. Monai: validation, formal analysis, writing – review & editing. R. Oord: investigation. J. E. Schmidt: methodology, supervision. E. T. C. Vogt*: validation, writing – review & editing. J. D. Poplawsky*: conceptualization, methodology, investigation, formal analysis, writing – review & editing. B. M. Weckhuysen*: conceptualization, validation, supervision, writing – review & editing.

## Conflicts of interest

There are no conflicts to declare.

## Supplementary Material

CY-012-D1CY01938D-s001
