# Supplementary material for: Nano-scale insights regarding coke formation in zeolite SSZ-13 subject to the methanol-to-hydrocarbons reaction
Source: Catal Sci Technol. 2022 Jan 8;12(4):1220–8. doi: 10.1039/d1cy01938d (PMC8859524; doi:10.1039/d1cy01938d)
Supplement: CY-012-D1CY01938D-s001 [file CY-012-D1CY01938D-s001.pdf]

Electronic Supplementary Material (ESI) for Catalysis Science & Technology.
This journal is © The Royal Society of Chemistry 2022

Nano-scale Insights Regarding Coke Formation in Zeolite SSZ-13 Subject to the Methanol-to-Hydrocarbons Reaction

S.H. van Vreeswijk[a] M. Monai[a], R. Oord[a], J.E. Schmidt[a], E.T.C. Vogt,[a,*] J.D. Poplawsky[b,*] and B.M. Weckhuysen[a,*]

[a] Inorganic Chemistry and Catalysis group, Debye Institute for Nanomaterials Science, Utrecht University, Universiteitsweg 99, 3854 CG Utrecht, The Netherlands.

[b] Center for Nanophase Materials Sciences, Oak Ridge National Laboratory, Oak Ridge, TN 37831, USA

S1 In-situ UV-vis spectroscopy

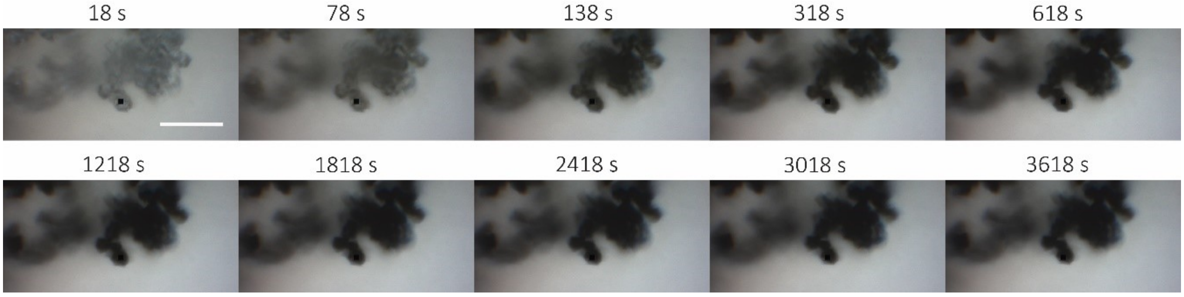

Figure S1: Optical microphotographs of the SSZ-13 crystals during the methanol-to-hydrocarbons process.

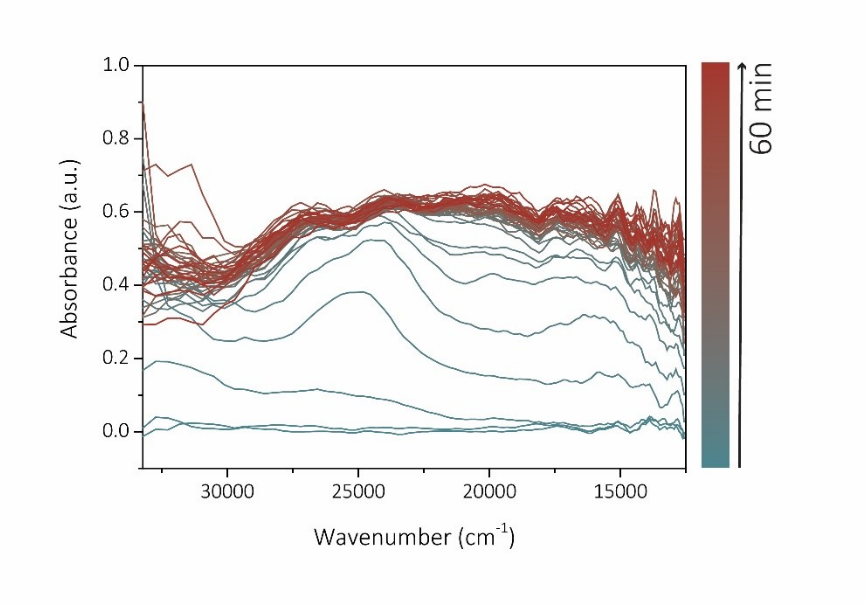

Figure S2: In-situ UV-vis spectra from 18 s to 60 min. A moving average has been used on the absorbance values for smoothing purposes.

S2 Atom probe tomography needle preparation

Figure S3 shows a schematic representation and SEM images of the needle preparation process.

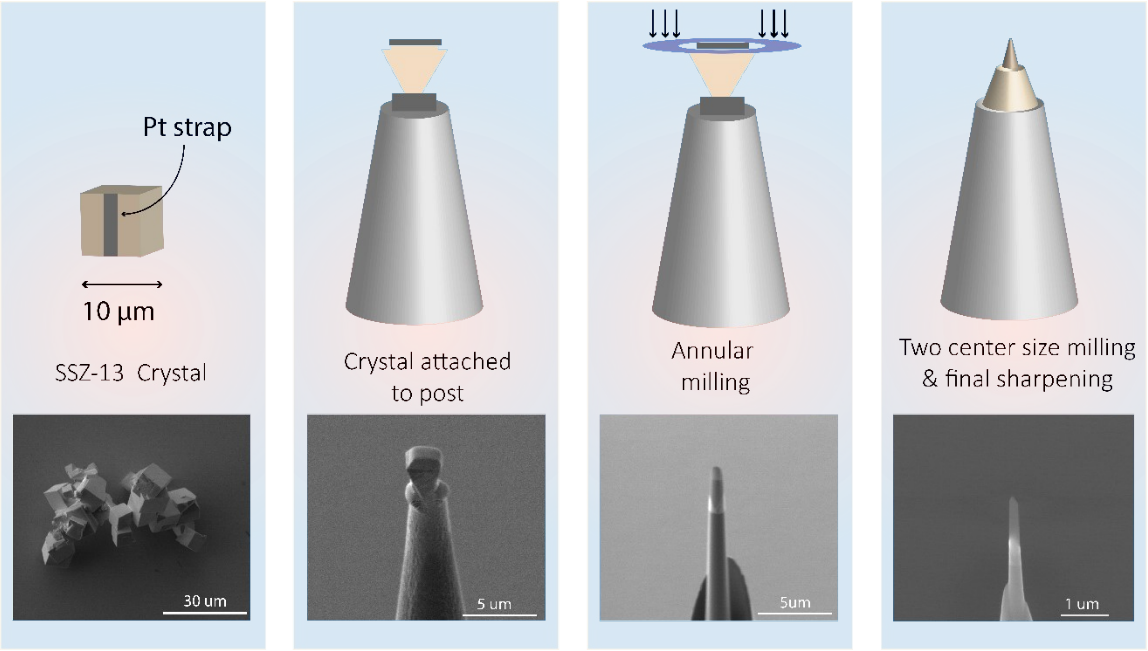

Figure S3: Schematic representation and scanning electron microscopy images of each step of the needle preparation method.

S3 Atom probe tomography data

S3.1 Atom maps, composition tables and TGA experiments

Unfortunately, not all atoms are evaporated as single ions during the APT measurements, especially because of the presence of ionically bonded elements such has oxygen and carbon in the zeolitic samples and evaporation often happens in the form of molecular ions. To be able to obtain information on the single atom level, the molecular ions can be decomposed. However, this means that the location of the single ions becomes more uncertain and therefore statics become very important in APT research. The composition tables below are showing the number of atom counts and the atomic percent in which these atoms appear. The presence of Ga is due to the Fib-milling steps (Ga-beam). The Si/Al ratios are depicted. The determined Si/Al ratio with ICP-OES is 18.8 and this thereby shows the consistency and credibility of the APT measurements. **Table S2** shows a slightly higher Si/Al ratio for H-SSZ-13 1 min coked, which could be due to the variations between crystals or within the crystal.

Table S 1: Composition of 0.5 min TOS needles (1,2)

**H-SSZ-13 0.5 min needle 1**			**H-SSZ-13 0.5 min needle 2**		
Ion	Count	Atomic %	Ion	Count	Atomic %
O	172923	57.163%	O	425062	57.681%
Si	110724	36.602%	Si	269677	36.595%
^{13}C	9662	3.194%	^{13}C	21636	2.936%
H	4359	1.441%	H	11164	1.515%
Al	3623	1.198%	Al	8184	1.111%
C	908	0.300%	C	1056	0.143%
Ga	311	0.103%	Ga	139	0.019%
	Si/Al	30.6		Si/Al	33.0

Table S 2: Composition of 1 min TOS needle (1)

**H-SSZ-13 1 min needle 1**		
Ion	Count	atomic %
O	624947	60.712%
Si	382145	37.124%
^{13}C	9508	0.924%
Al	8372	0.813%
Ga	3407	0.331%
C	967	0.094%
	Si/Al	45.6

Table S 3: Composition of 3 min TOS needles (1,2)

**H-SSZ-13 3 min needle 1**			**H-SSZ-13 3 min needle 2**		
Ion	Count	Atomic %	Ion	Count	Atomic %
O	776088	55.205%	O	1230388	57.902%
Si	494369	35.165%	Si	639883	30.113%
^{13}C	111378	7.922%	^{13}C	190775	8.978%
Al	21278	1.514%	H	30912	1.455%
Ga	1606	0.114%	Al	23777	1.119%

C	1122	0.080%	C	6075	0.286%
Bk	0	0.000%	Ga	3099	0.146%
	Si/Al	23.2		Si/Al	26.9

Table S 4: Composition of 5 min TOS needles (1,2,3)

H-SSZ-13 5 min needle 1			H-SSZ-13 5 min needle 2			H-SSZ-13 5 min needle 3		
Ion	Count	Ion	Ion	Count	Atomic %	Ion	Count	Atomic %
O	410846	56.214%	O	471146	54.373%	O	277917	54.398%
Si	247354	33.844%	Si	256006	29.545%	Si	157678	30.863%
^{13}C	61164	8.369%	^{13}C	113715	13.123%	^{13}C	56237	11.007%
Al	10312	1.411%	H	15816	1.825%	H	12182	2.384%
C	527	0.072%	Al	9188	1.060%	Al	6304	1.234%
Ga	462	0.063%	C	395	0.046%	Ga	346	0.068%
Bk	193	0.026%	Ga	235	0.027%	C	194	0.038%
	Si/Al	24.0		Si/Al	27.9		Si/Al	25.0

Table S 5: Composition of 15 min TOS needle (1)

H-SSZ-13 15 min needle 1		
Atom	Count	Ranged %
O	73837	49.20%
Si	41673	27.80%
^{13}C	26581	17.70%
H	4628	3.10%
Al	2121	1.40%
^{12}C	734	0.50%
Ga	557	0.40%
	Si/Al	19.6

Table S 6: Composition of 30 min TOS needles (1,2)

H-SSZ-13 30 min needle 1			H-SSZ-13 30 min needle 2		
Ion	Count	Ranged %	Ion	Count	Ranged %
O	207037	51.40%	O	919899	52.00%
Si	114064	28.30%	Si	522196	29.50%
^{13}C	62723	15.60%	^{13}C	264384	14.90%
H	11977	3.00%	H	34850	2.00%
Al	4697	1.20%	Al	19688	1.10%
C	1703	0.40%	C	5467	0.31%
			Ga	2670	0.15%
	Si/Al	24.28		Si/Al	26.5

Table S 7: Composition of 60 min TOS needles (1,2)

H-SSZ-13 60 min needle 1			H-SSZ-13 60 min needle 2		
Ion	Count	Ranged %	**Ion**	21487	1.57%
O	1460519	50.40%	O	2283804	51.065%
Si	717246	24.70%	Si	1370127	30.636%
^{13}C	620721	21.40%	^{13}C	681038	15.228%
H	57714	2.00%	H	82489	1.844%
Al	28185	0,972%	Al	42686	0.954%
Ga	1792	0,062%	C	2382	0.053%
	Si/Al	25.4		Si/Al	32.1

Table S 8: Composition of 120 min TOS needles (1,2)

H-SSZ-13 120 min needle 1			H-SSZ-13 120 min needle 2		
Al	6897	1.45%	Al	21487	1.57%
O	250146	52.48%	O	707026	51.63%
Ga	4505	0.95%	Ga	13475	0.98%
Si	112925	23.69%	Si	346852	25.33%
12C	18824	3.95%	C	45003	3.29%
^{13}C	82003	17.20%	^{13}C	228939	16.72%
H	1331.885	0.28%	H	6575	0.48%
	Si/Al	18.71032		Si/Al	17.70963

When all the obtained mass spectra are analyzed the needles can be reconstructed, because the location is determined with the position sensitive detector. Atom maps of the relevant needles of the relevant atoms are depicted in the next figures in this section. The ion maps before Ga subtraction are depicted for clarity. For the solute analysis (NND, RDF), the Ga-damage has been subtracted.

To be able to assure the reliability of the amount of coke measured with APT, the data is, besides to the UV-vis spectroscopy experiments, compared to the amount of coke found with thermogravimetric analysis / mass spectrometry of the fully coked samples measured with the *operando* UV-vis set-up after 180 min (**Figure S4**). It was found that indeed the catalyst looses about 18% coke upon oxidation, which is in total agreement with the found APT results for the fully coked samples (15 to 120 min).

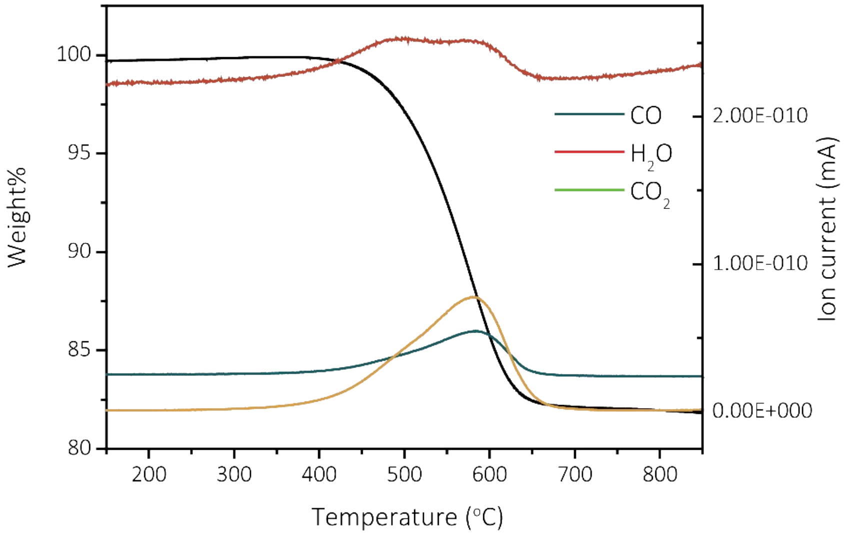

Figure S4: Thermogravimetric analysis / mass spectrometry of the fully coked SSZ-13 sample after reaction in the *operando* UV-vis spectroscopy set-up (180 min).

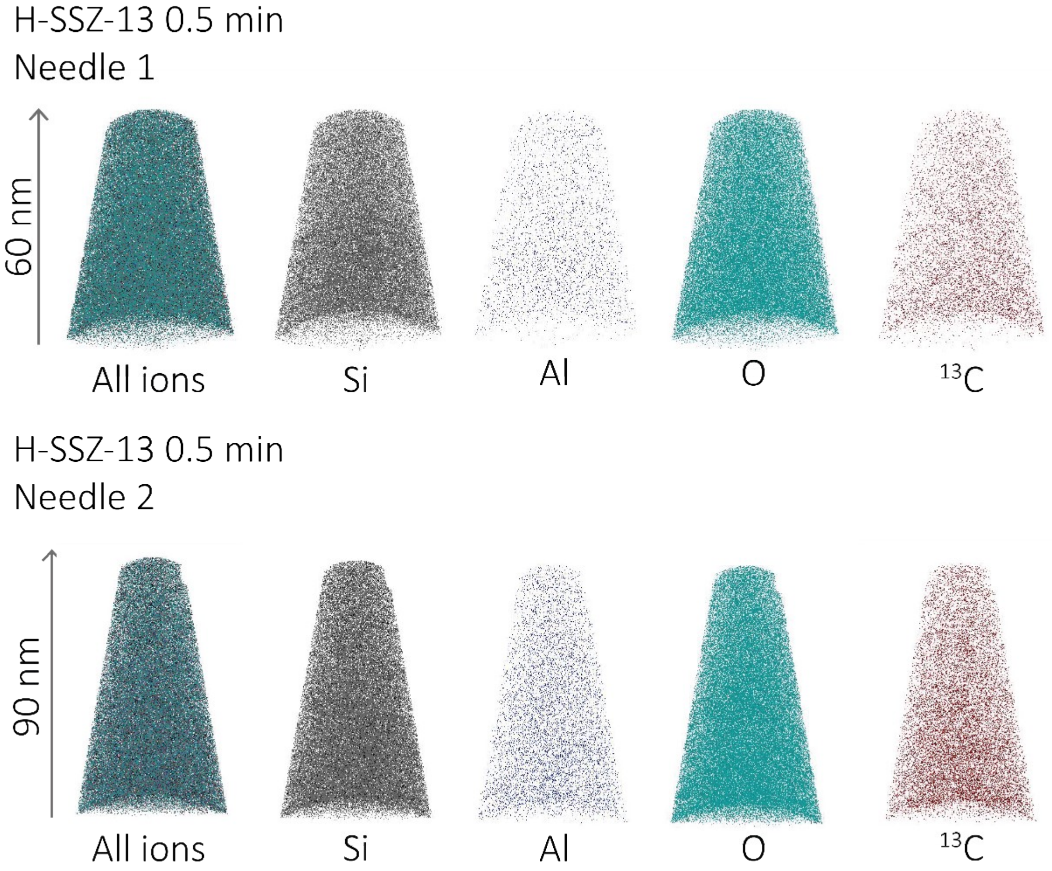

Figure S5: Needle reconstruction of H-SSZ-13 0.5 min time-on-stream.

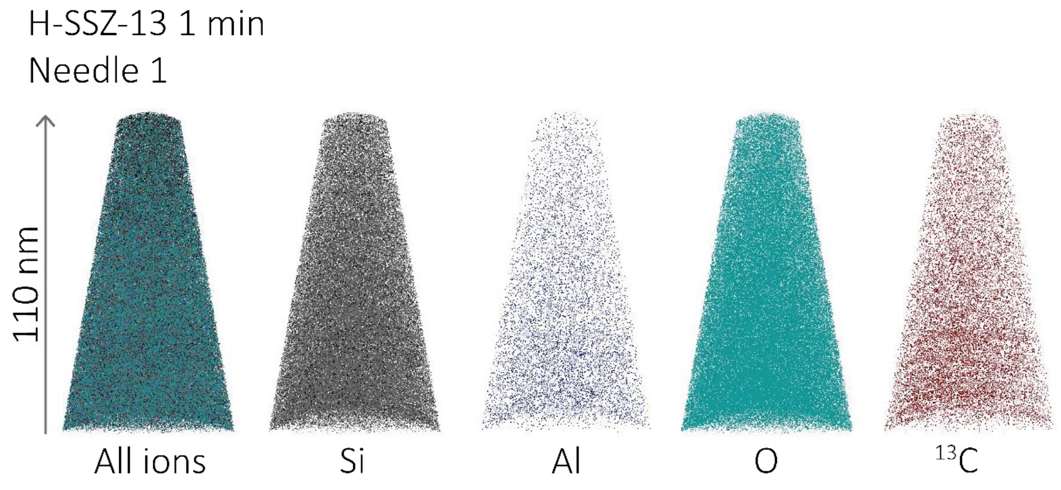

Figure S6: Needle reconstruction of H-SSZ-13 1 min time-on-stream.

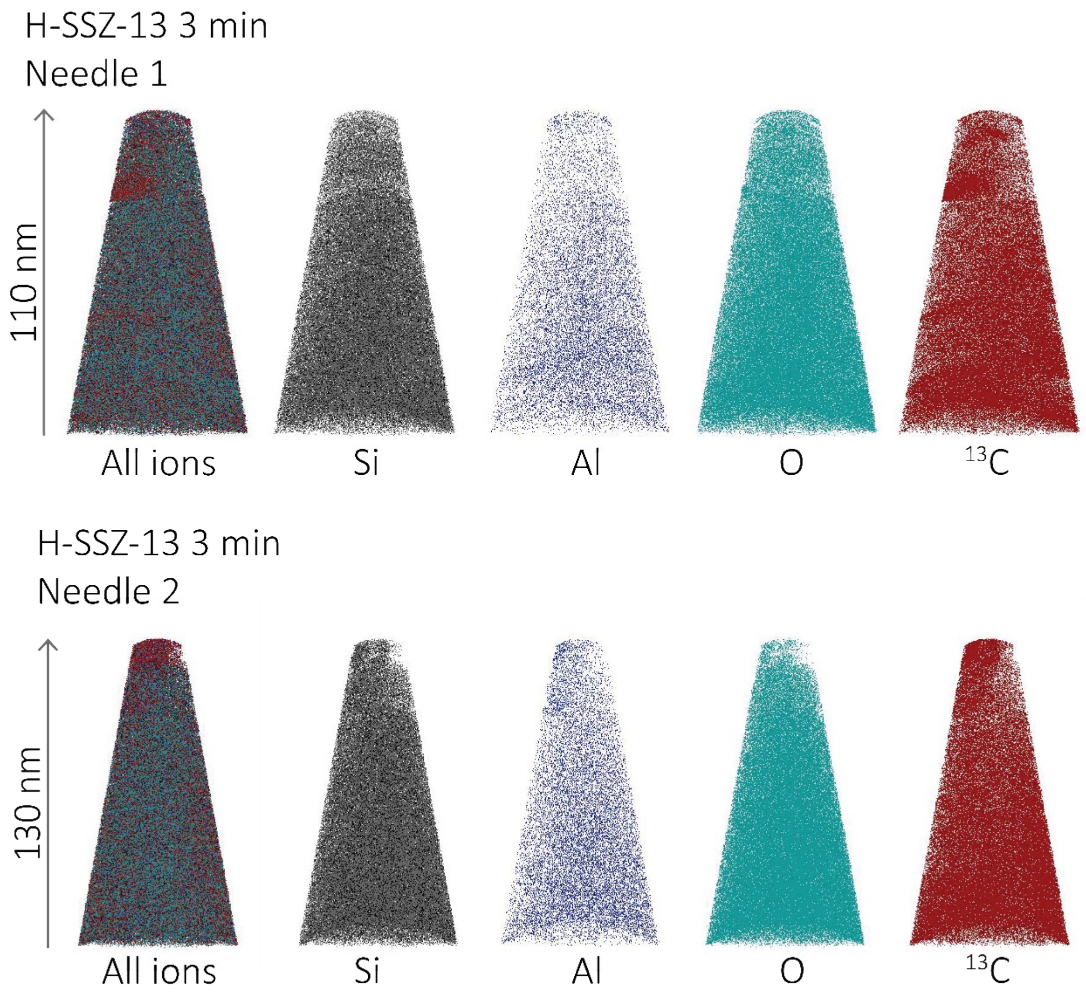

Figure S7: Needle reconstruction of H-SSZ-13 3 min time-on-stream.

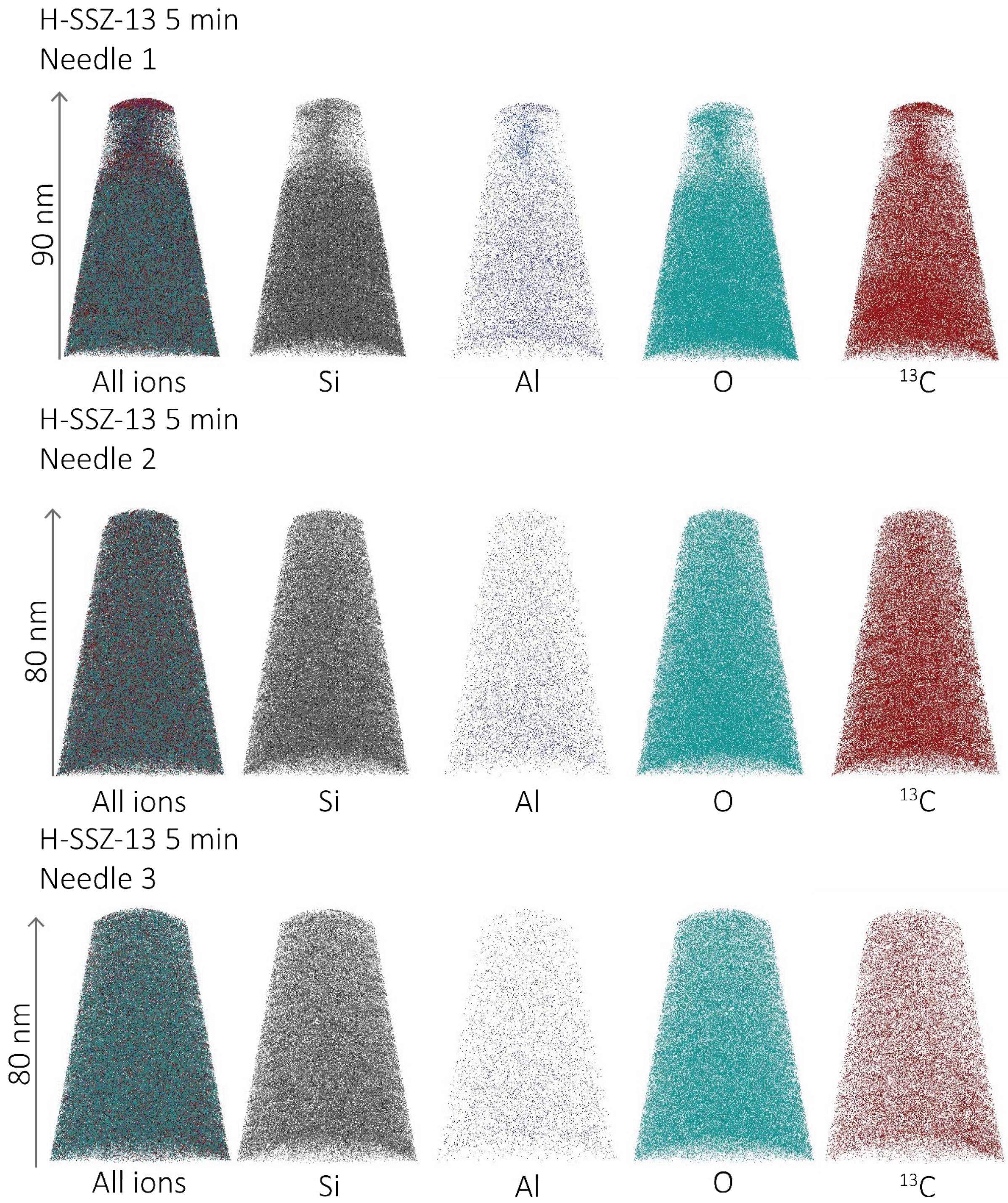

Figure S8: Needle reconstruction of H-SSZ-13 5 min time-on-stream.

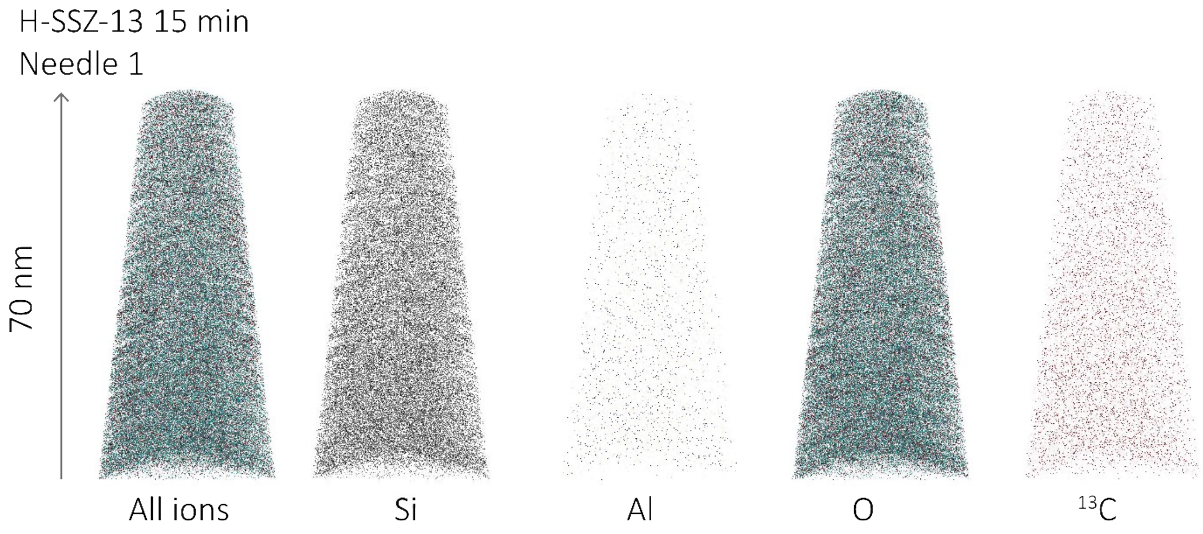

Figure S9: **Needle reconstruction of H-SSZ-13 15 min time-on-stream.**

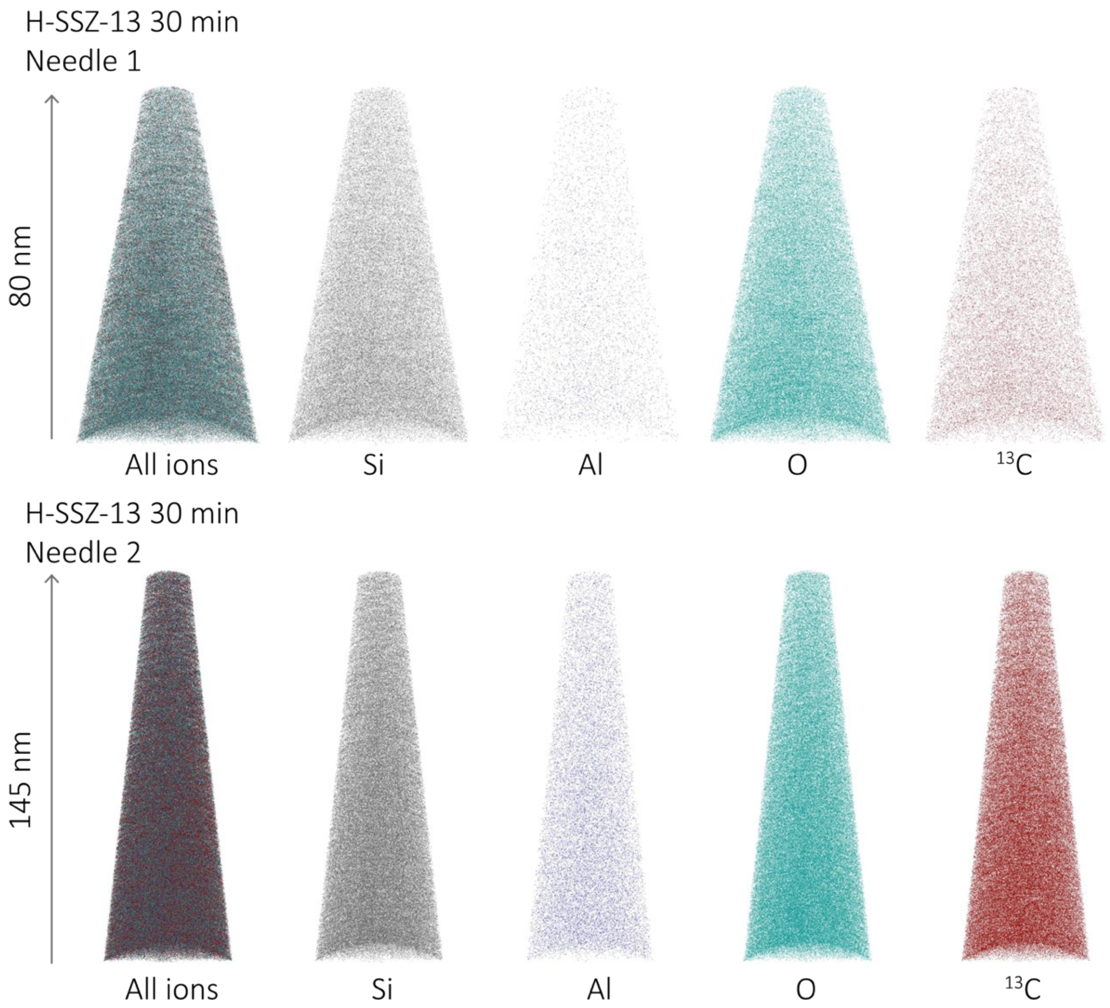

Figure S10: **Needle reconstruction of H-SSZ-13 30 min time-on-stream.**

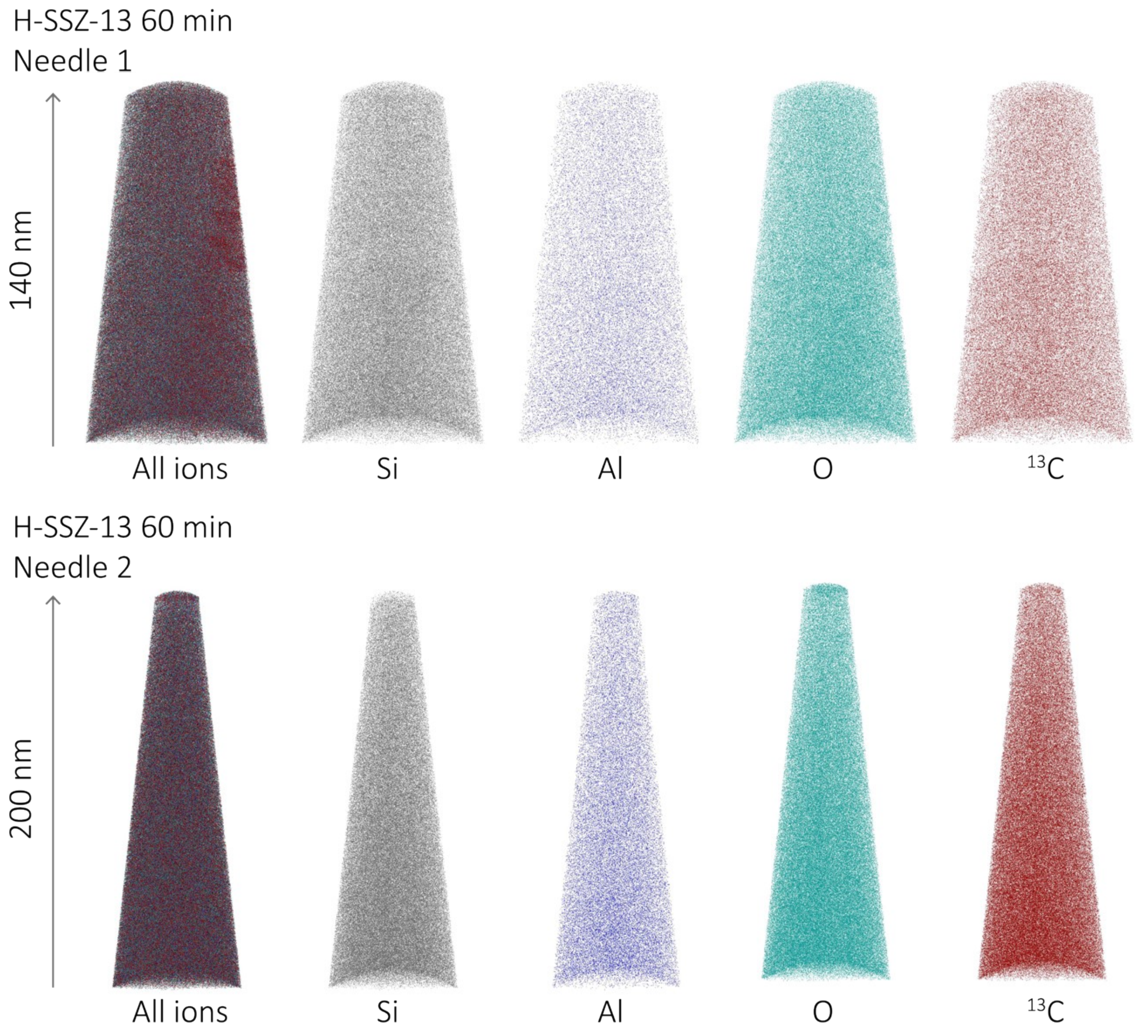

Figure S11: **Needle reconstruction of H-SSZ-13 60 min time-on-stream.**

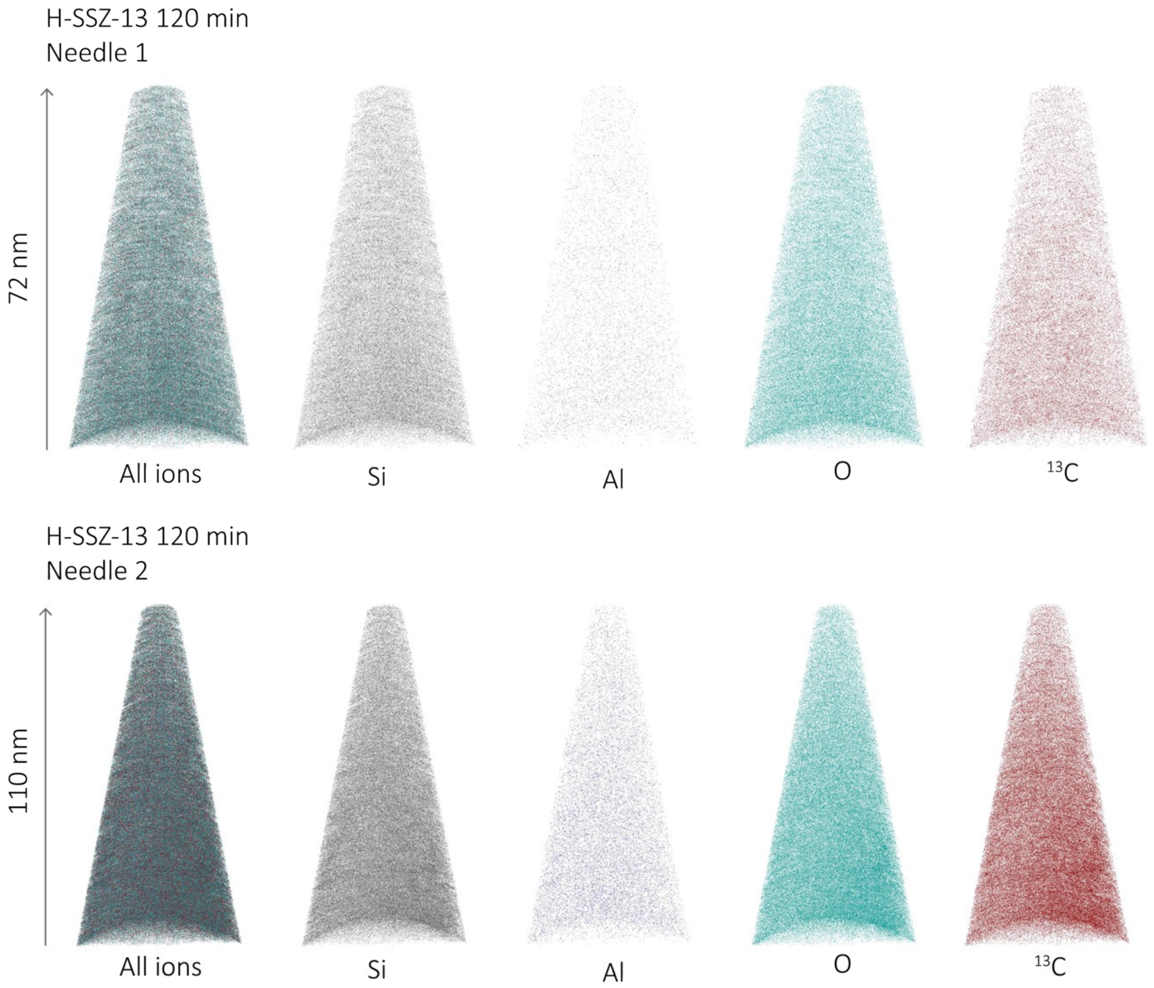

Figure S12: Needle reconstruction of H-SSZ-13 120 min time-on-stream.

S3.2 Iso-surface analysis

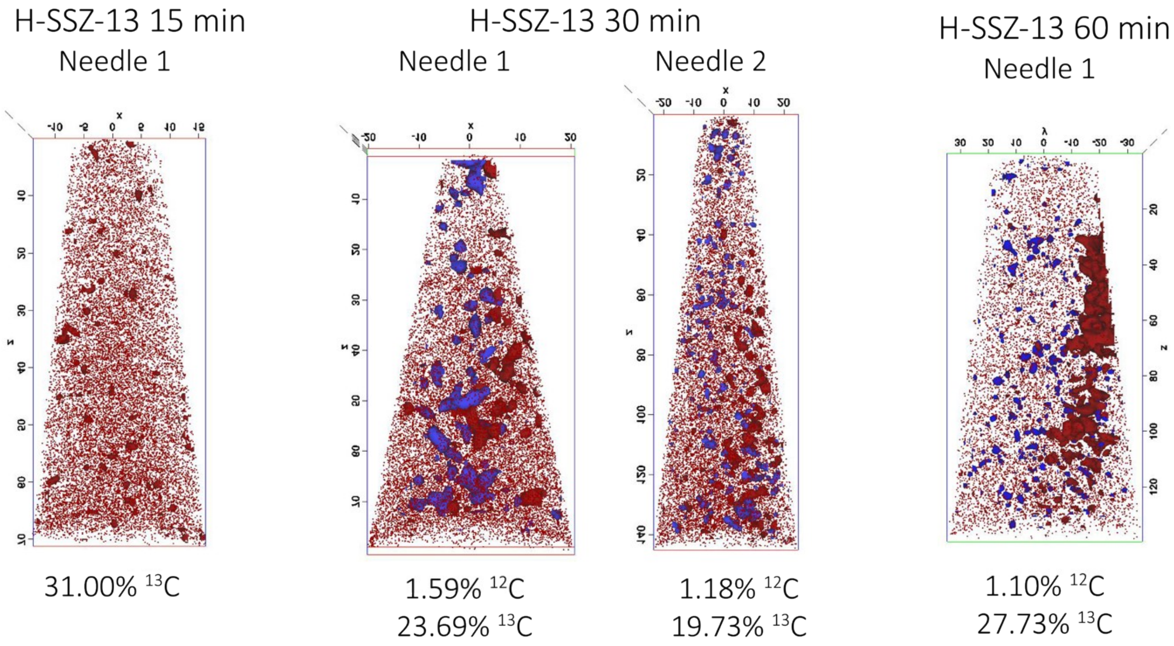

Figure S13: Iso-surface analysis of some selected needles. Indications of carbon rich and pore areas are shown.

S3.3 Nearest neighbour distribution

The nearest neighbor distribution (NND) analysis can be used to analyze the distribution of the species of interest. A random nearest neighbor distribution is simulated by randomly placing the number of atoms counted in the volume of the needle measured and measuring the distance between these atoms. With enough counts, this results in a random normal distribution (Gaussian curve) with the atom pair distance on the x-axis and the number of counts on the y-axis. When elements cluster, the nearest neighbor distribution has a multimodal distribution with the distances of the clustered and non-clustered atoms embedded in the curve. This can cause the main peak to shift to lower atom pair distances.[1,2,3,4] The analysis can be conducted for first order neighbors, but also for higher order neighbors which can help to remove random fluctuations in the data.[1,5] In this study, both first order NNDs and fifth order NNDs are presented.

The graphs with ^{13}C pair distances refer to the distance between the ^{13}C single atoms found in the mass spectrum. The graphs with ^{13}C (in molecules) give a more complete representation as all the molecules and atoms in which ^{13}C was present are taken into account. The NND of Si and Al represent the distribution of Si and Al found both as single atoms and molecules.

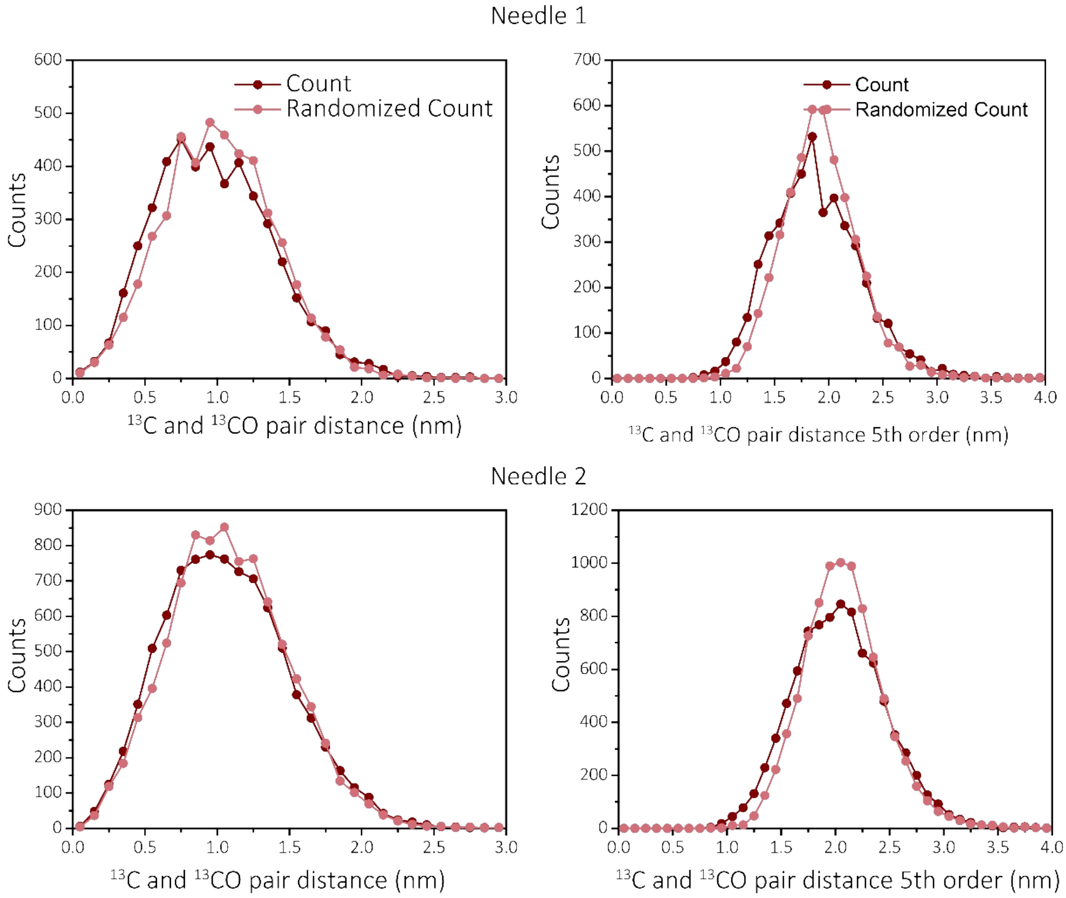

Figure S14: First and fifth order nearest neighbor distribution functions of the H-SSZ-13 0.5 min coked sample needle 1 and 2 of ^{13}C and ^{13}CO molecules/atoms found in the MS.

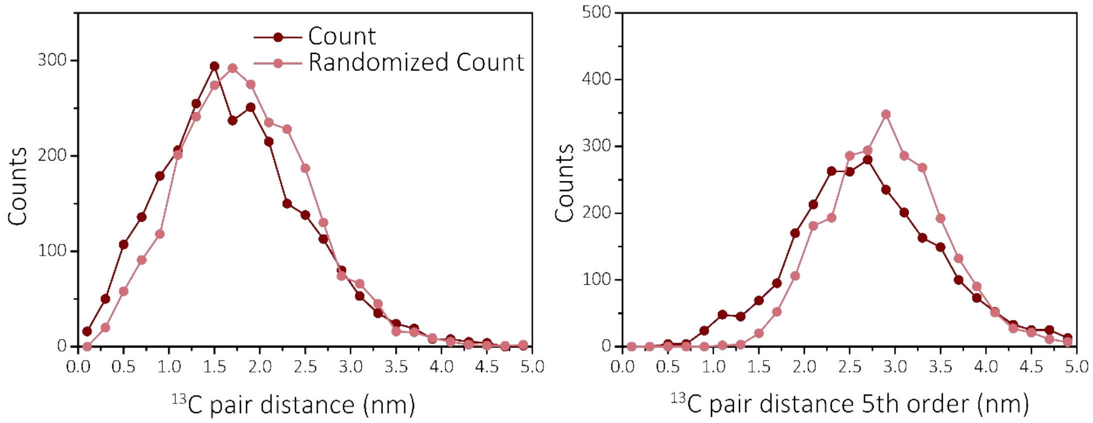

Figure S15: First and fifth order nearest neighbor distribution functions of the H-SSZ-13 1 min coked sample needle 1 and ^{13}C single atoms found in the MS and ^{13}C single atoms.

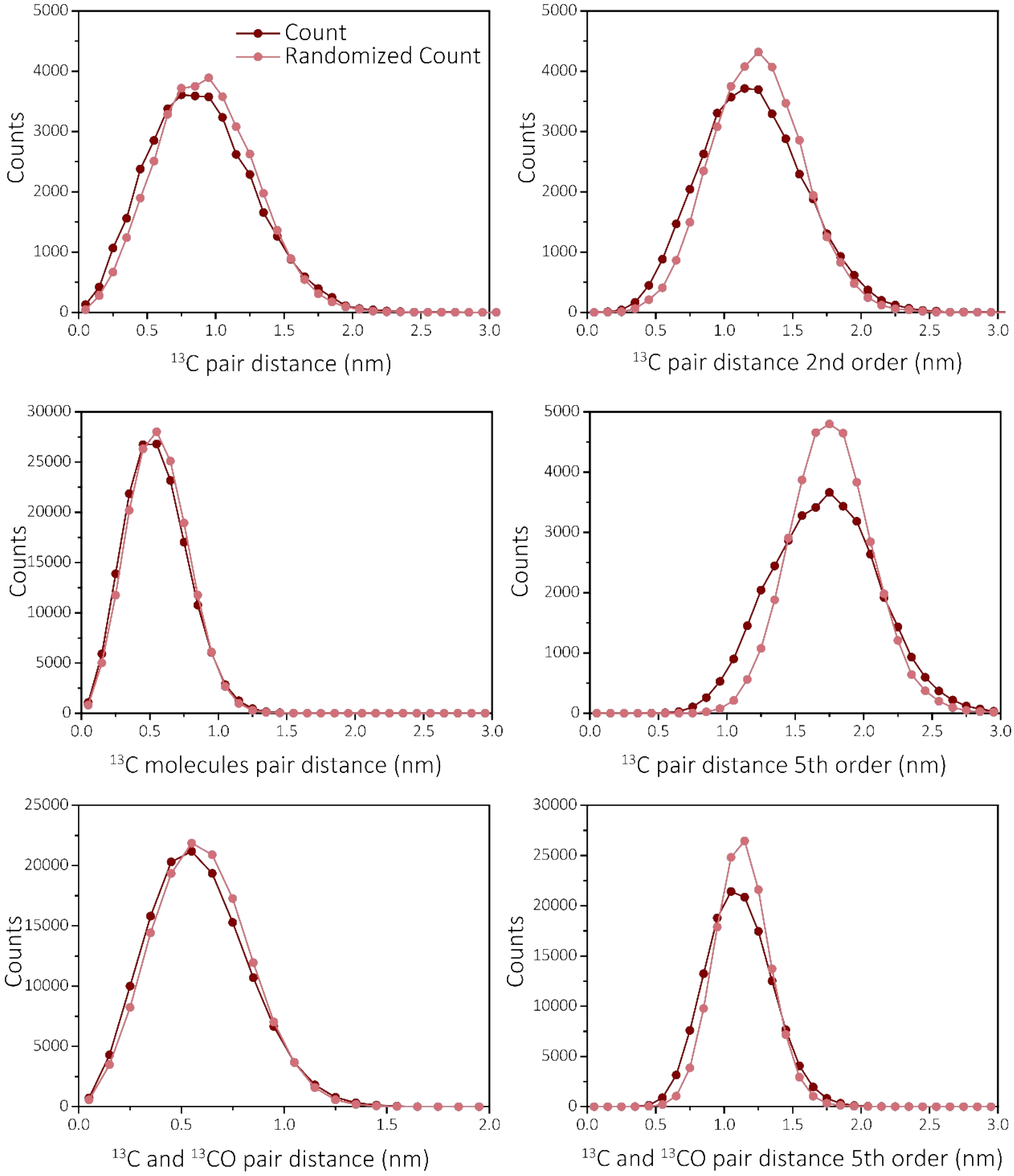

Figure S16: First and fifth order nearest neighbor distribution functions of the H-SSZ-13 3 min coked sample needle 2 of ^{13}C and ^{13}CO atoms/molecules found in the MS.j

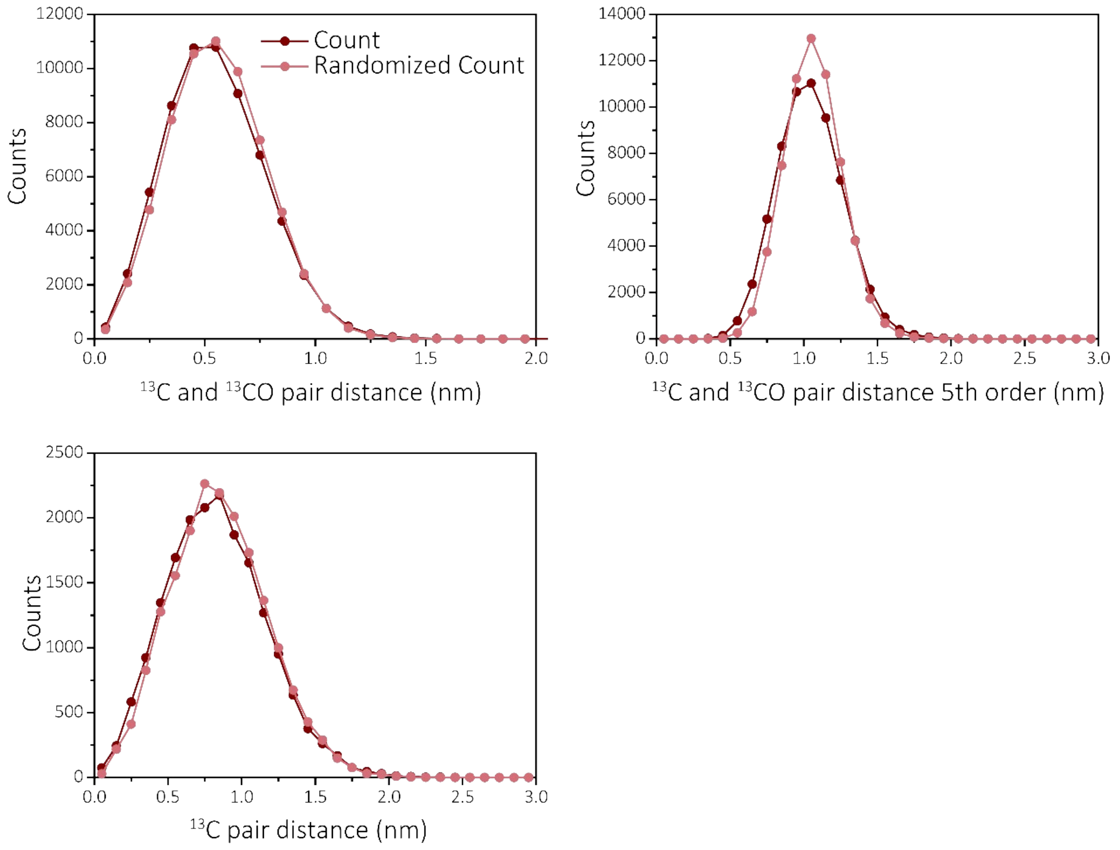

Figure S17: First and fifth order nearest neighbor distribution functions of the H-SSZ-13 5 min coked sample needle 1 of ^{13}C and ^{13}CO atoms/molecules found in the MS.

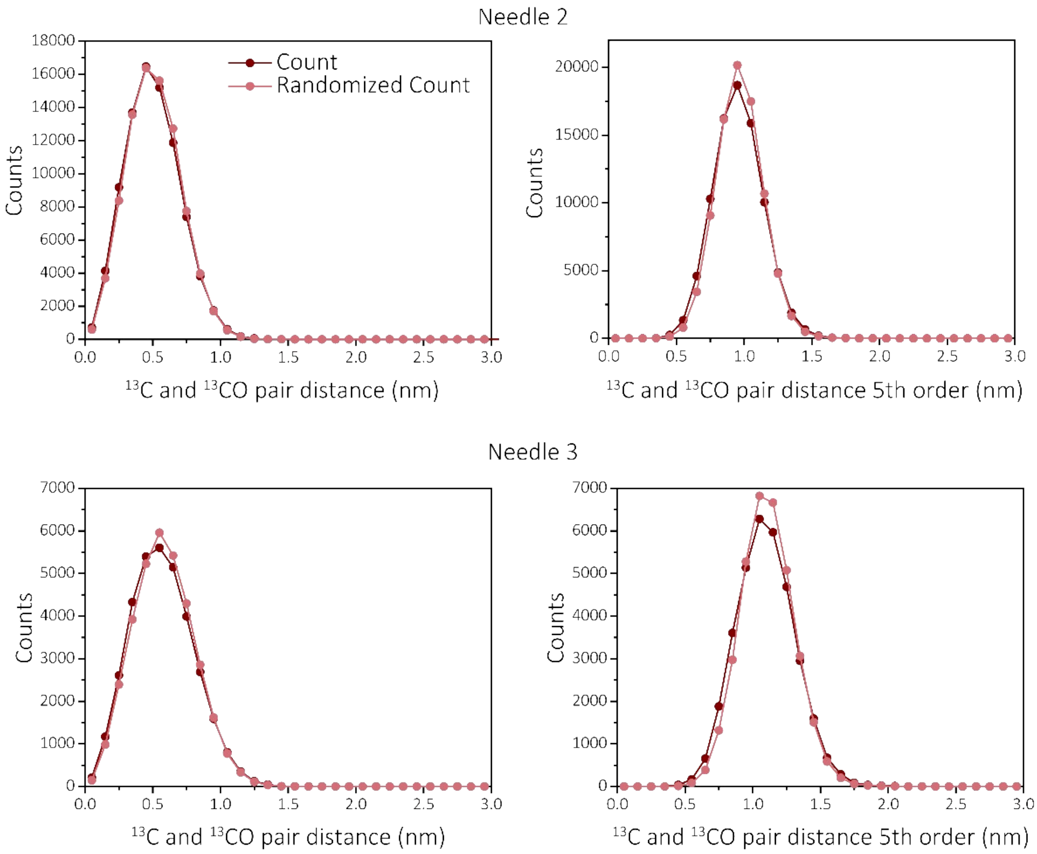

Figure S18: First and fifth order nearest neighbor distribution functions of the H-SSZ-13 5 min coked sample needle 2 and 3 of ^{13}C and ^{13}CO atoms/molecules found in the MS.

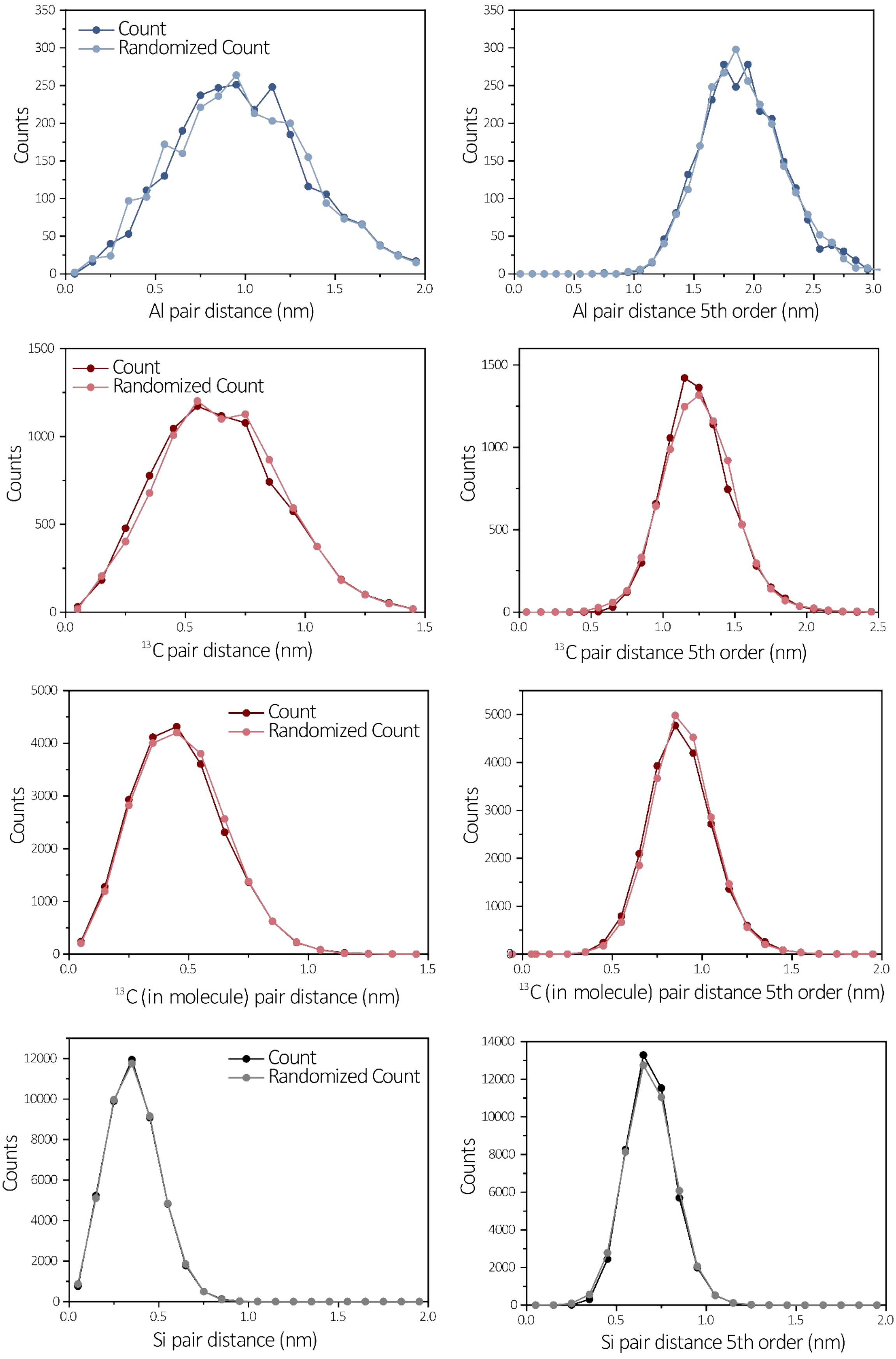

Figure S19: First and fifth order nearest neighbor distribution functions of the H-SSZ-13 15 min coked sample needle 1 of Si, Al and ^{13}C decomposed from the molecules found in the MS and ^{13}C single atoms.

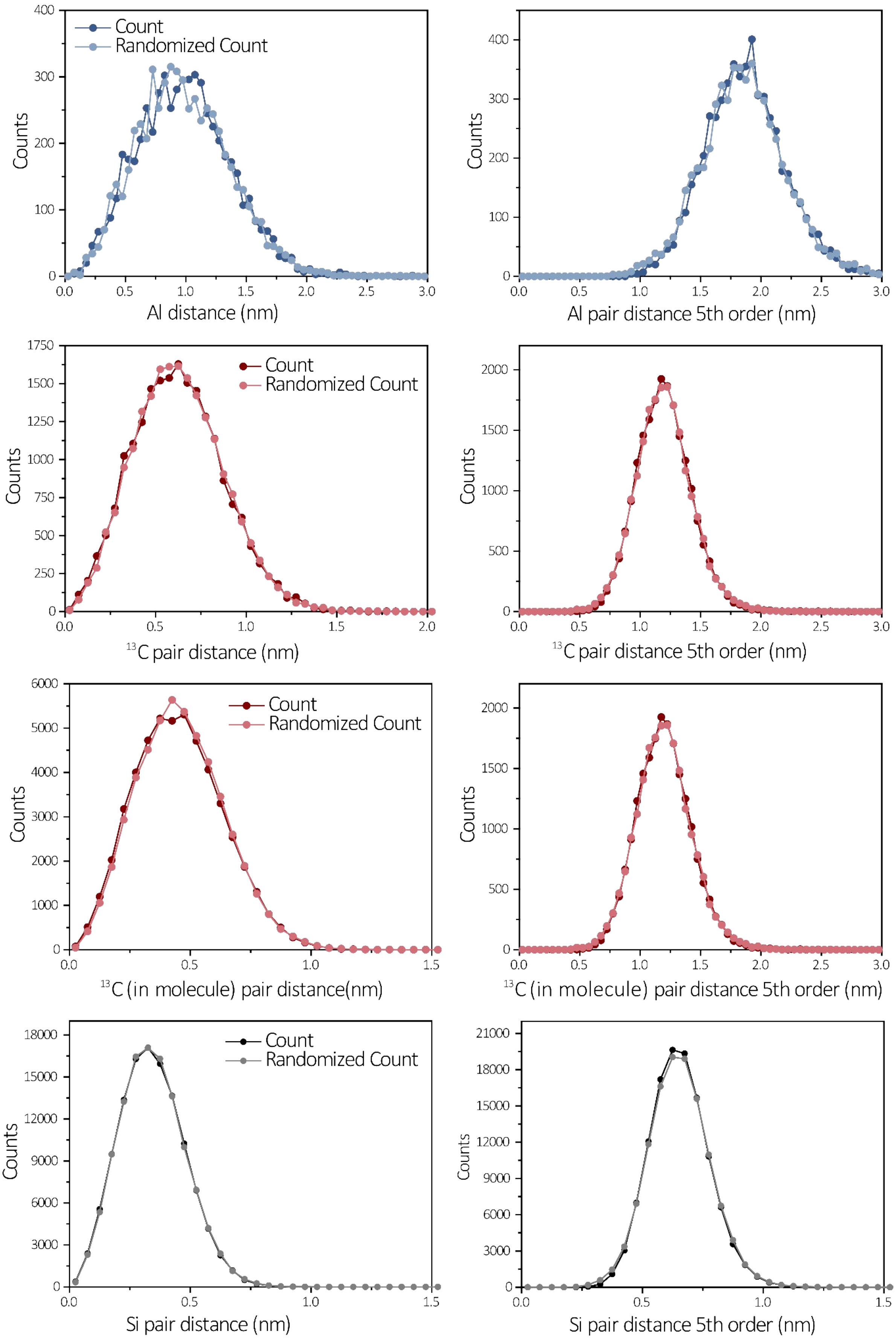

Figure S20: First and fifth order nearest neighbor distribution functions of the H-SSZ-13 30 min coked sample needle 1 of Si, Al and ^{13}C decomposed from the molecules found in the MS and ^{13}C single atoms.

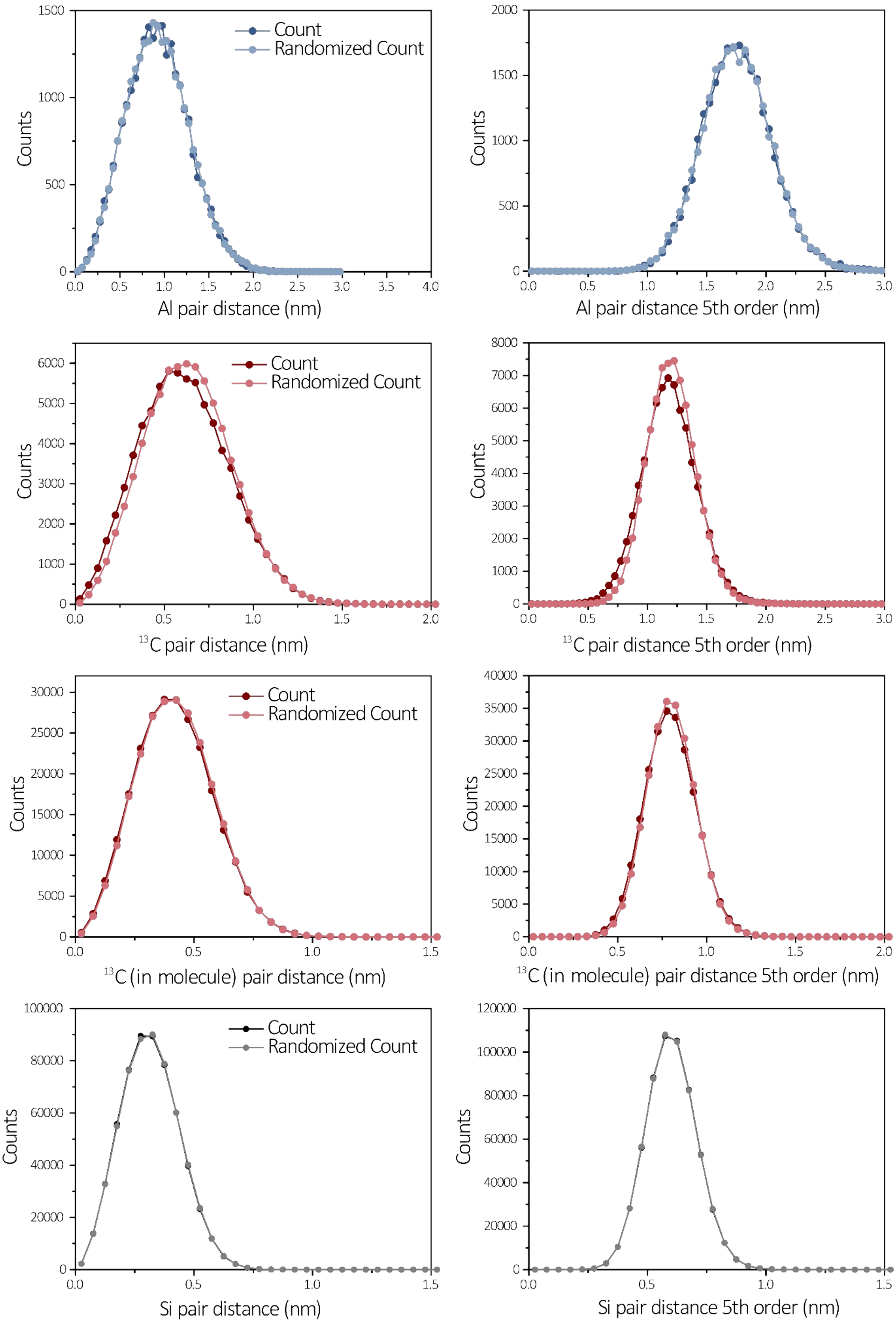

Figure S21: First and fifth order nearest neighbor distribution functions of the H-SSZ-13 30 min coked sample needle 2 of Si, Al and ^{13}C decomposed from the molecules found in the MS and ^{13}C single atoms.

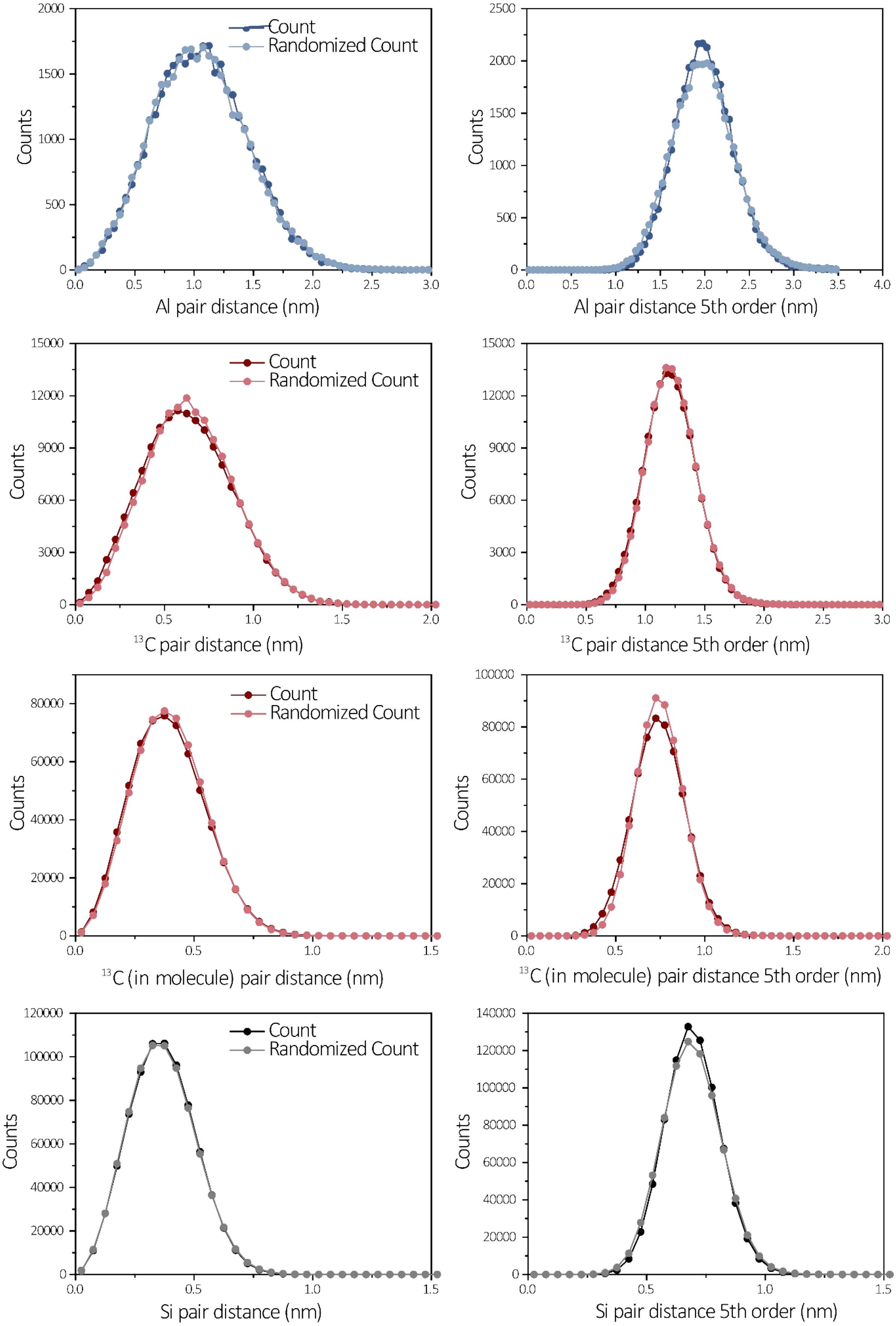

Figure S22: First and fifth order nearest neighbor distribution functions of the H-SSZ-13 60 min coked sample needle 1 of Si, Al and ^{13}C decomposed from the molecules found in the MS and ^{13}C single atoms.

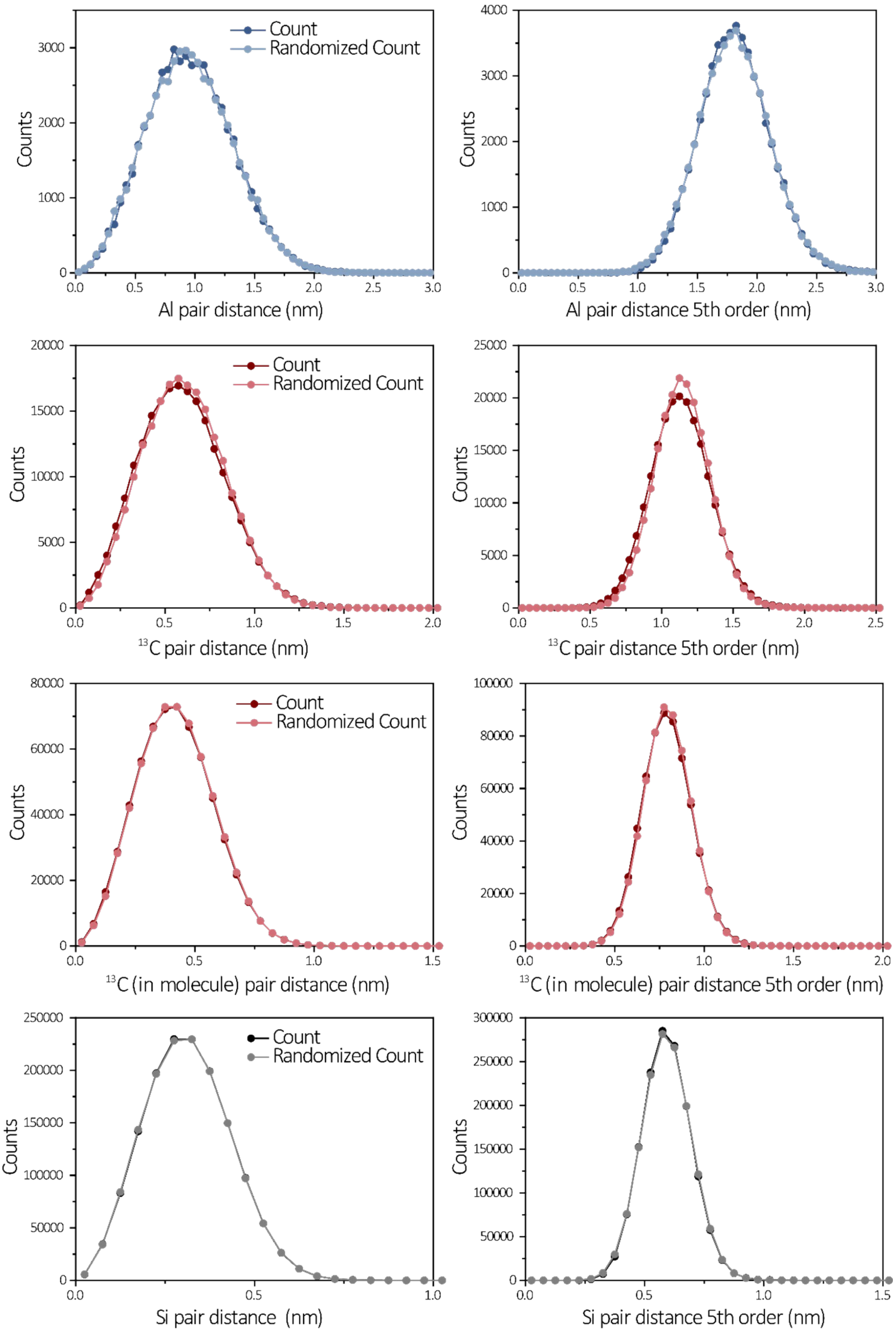

Figure S23: First and fifth order nearest neighbor distribution functions of the H-SSZ-13 60 min coked sample needle 2 of Si, Al and ^{13}C decomposed from the molecules found in the MS and ^{13}C single atoms.

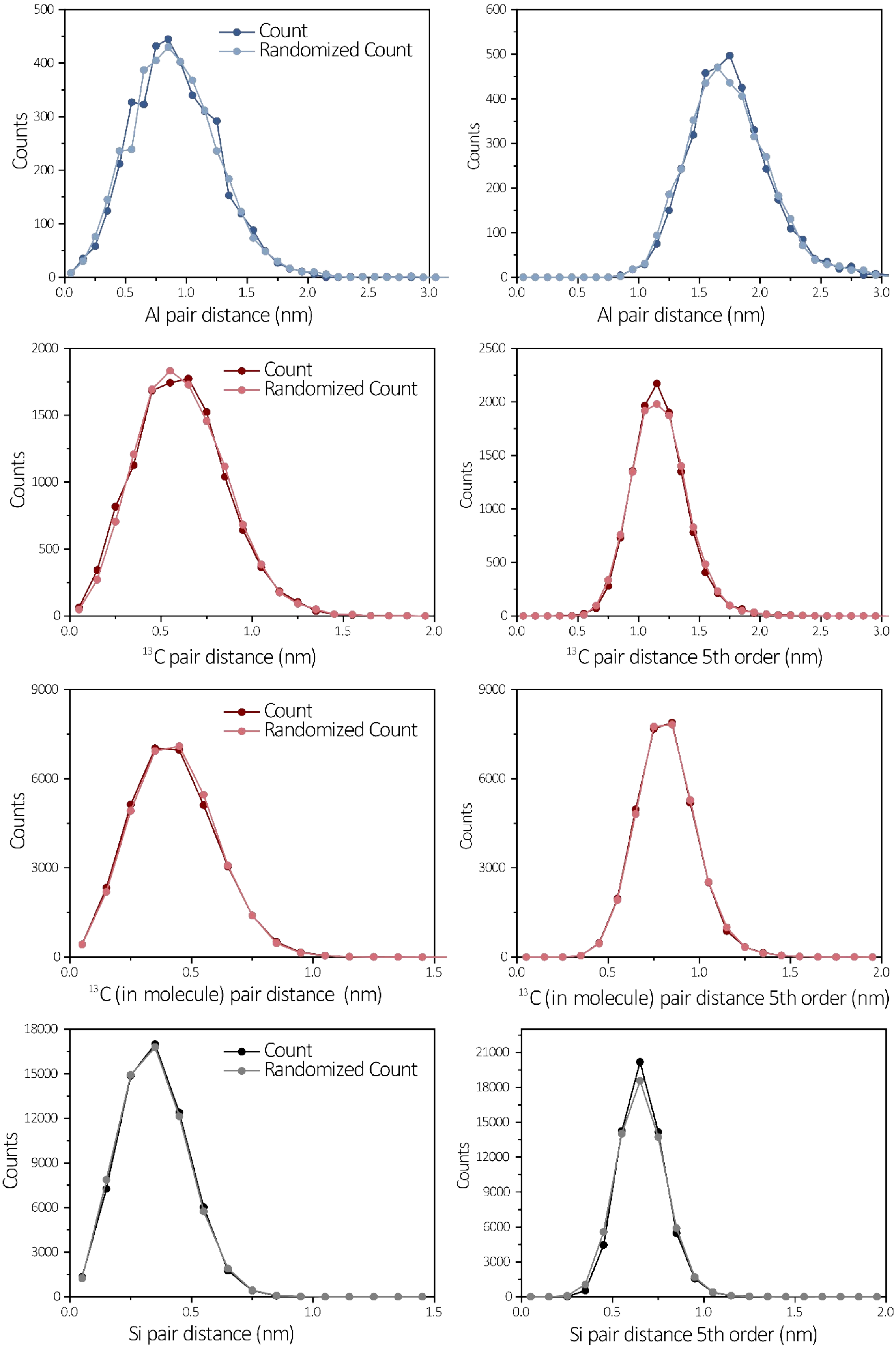

Figure S24: First and fifth order nearest neighbor distribution functions of the H-SSZ-13 120 min coked sample needle 1 Si, Al and ^{13}C decomposed from the molecules found in the MS and ^{13}C single atoms.

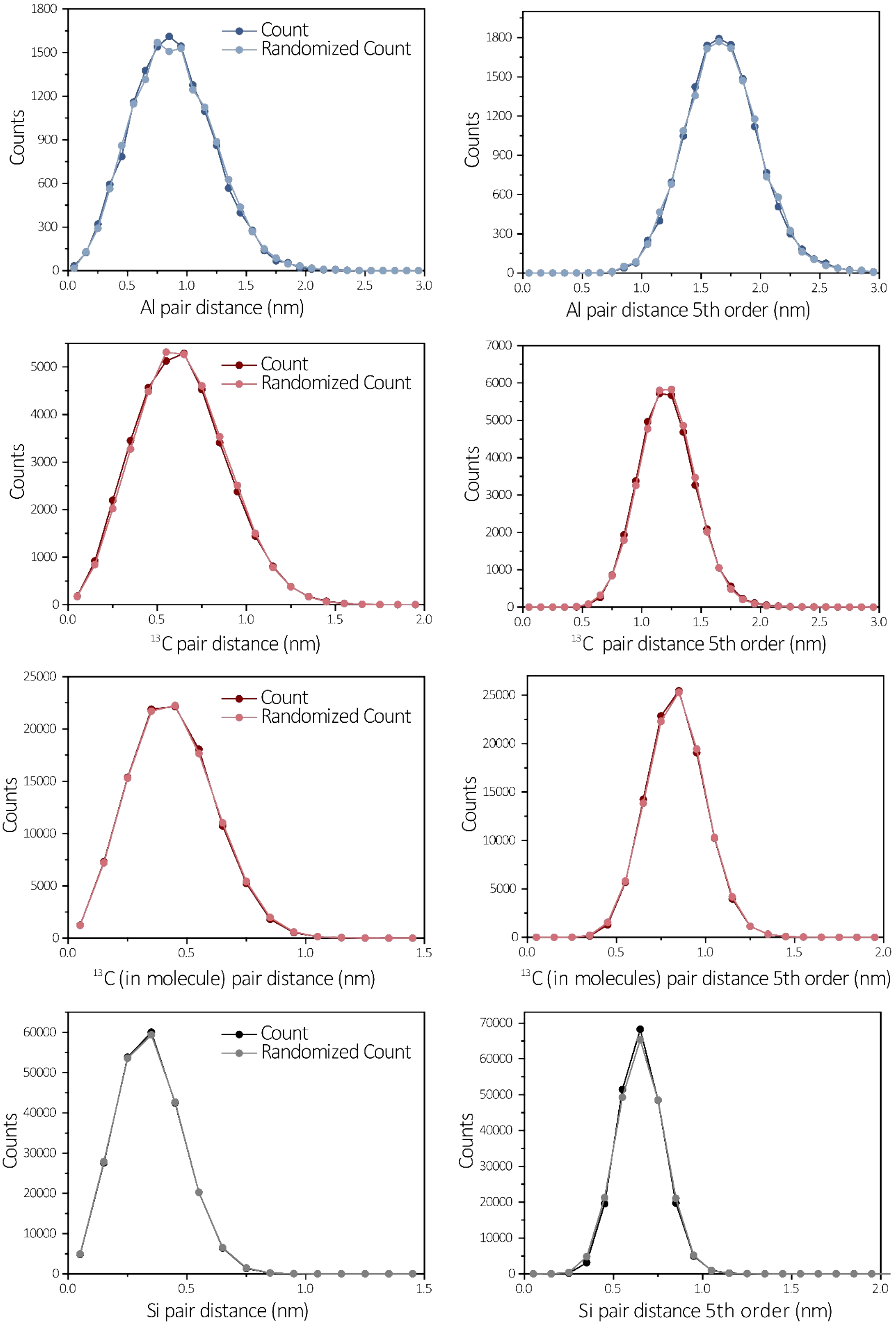

Figure S25: First and fifth order nearest neighbor distribution functions of the H-SSZ-13 120 min coked sample needle 2 of Si, Al and ^{13}C decomposed from the molecules found in the MS and ^{13}C single atoms.

S3.4 Nearest neighbor distribution analysis fitting

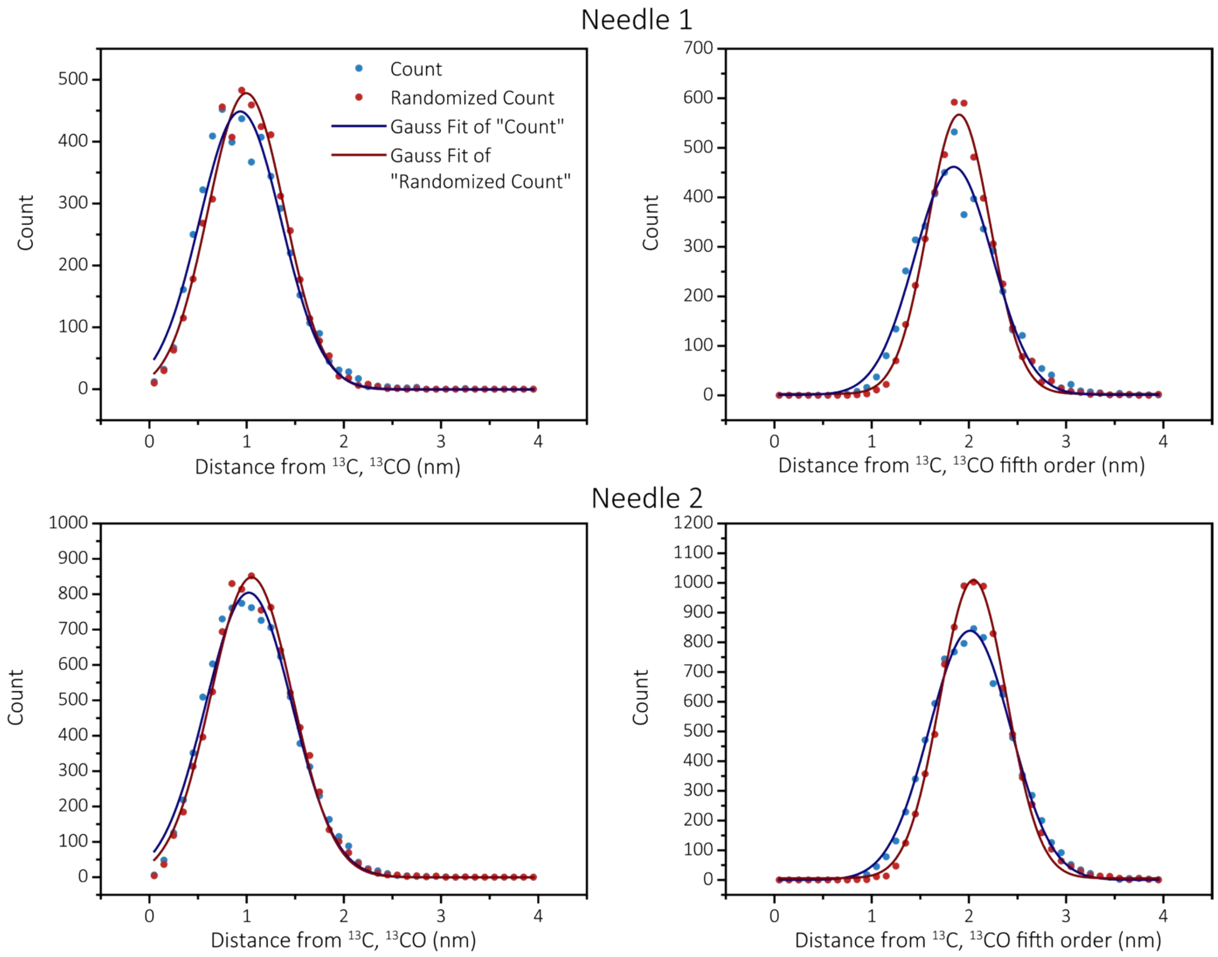

Figure S26: Fit of the randomized nearest neighbor distributions and measured nearest neighbor distributions of 0.5 min samples.

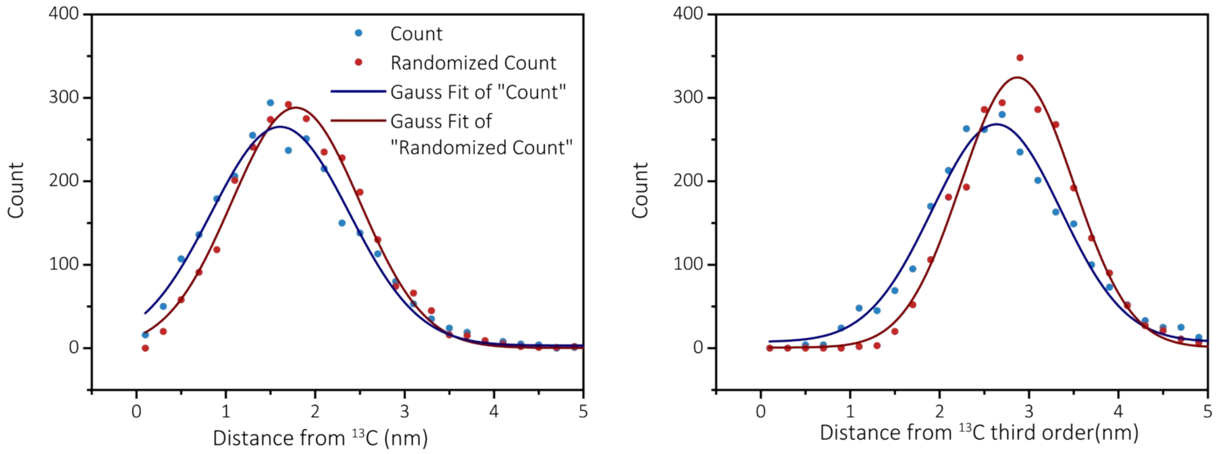

Figure S27: Fit of the randomized nearest neighbor distributions and measured nearest neighbor distributions of 1 min samples.

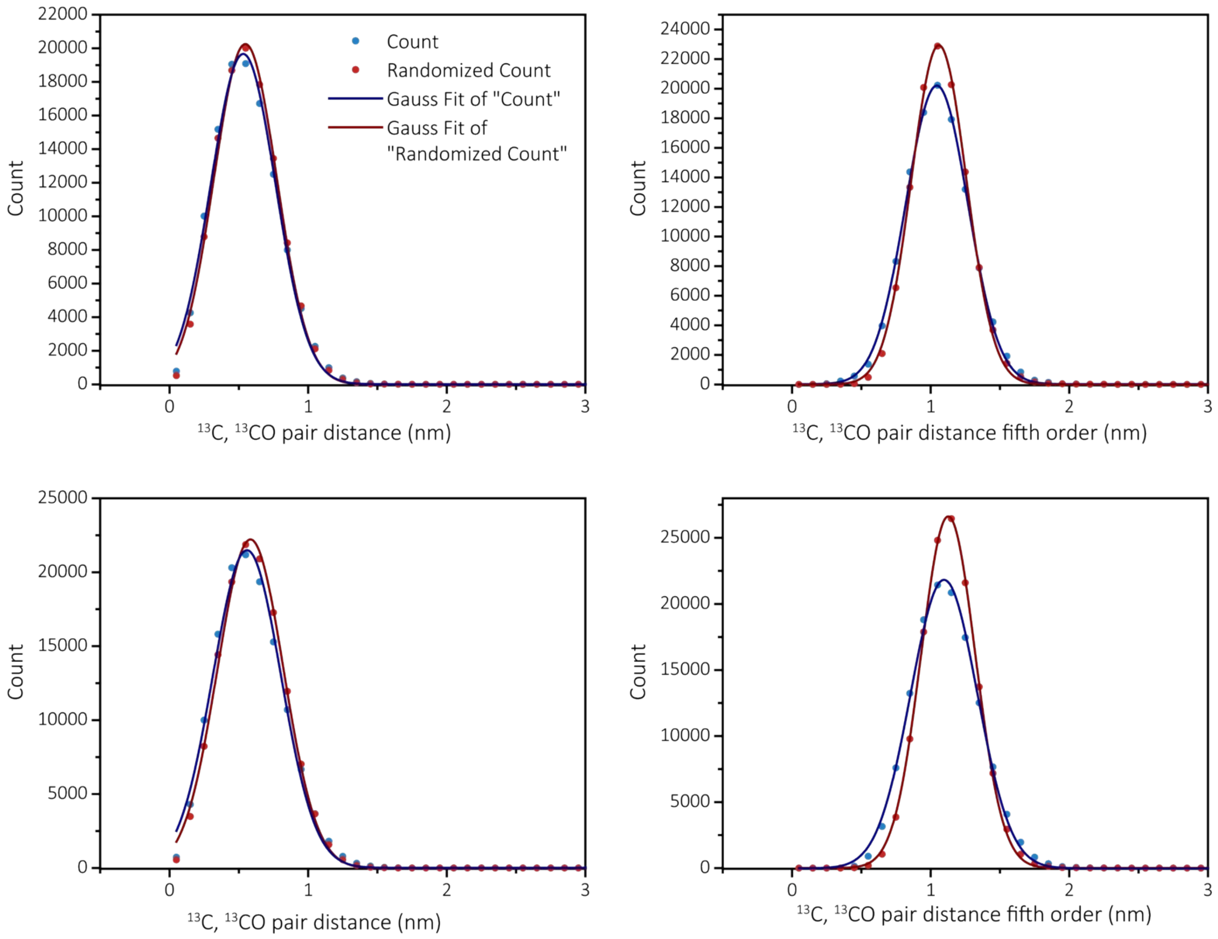

Figure S28: Fit of the randomized nearest neighbor distributions and measured nearest neighbor distributions of 3 min samples.

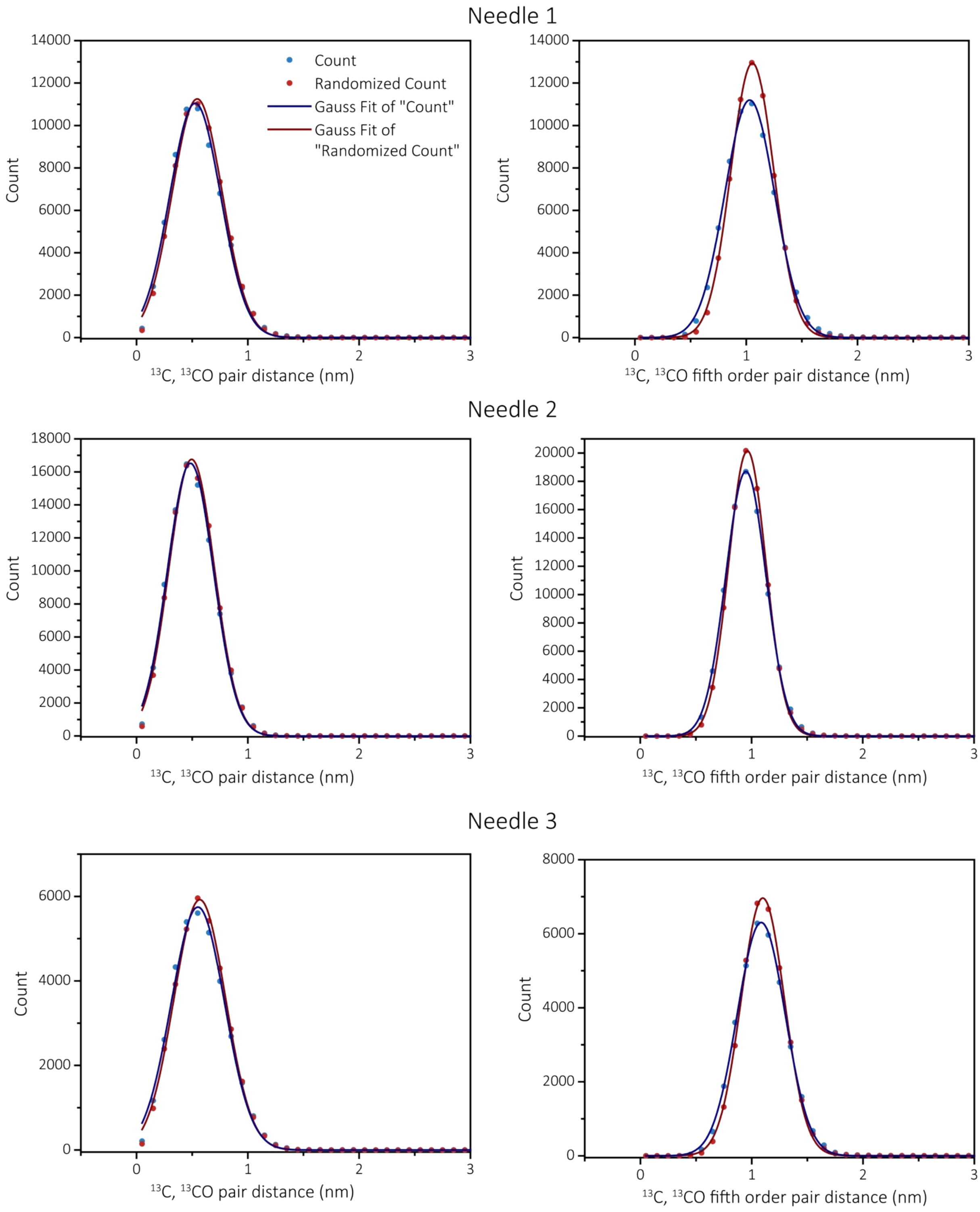

Figure S29: Fit of the randomized nearest neighbor distributions and measured nearest neighbor distributions of 5 min samples.

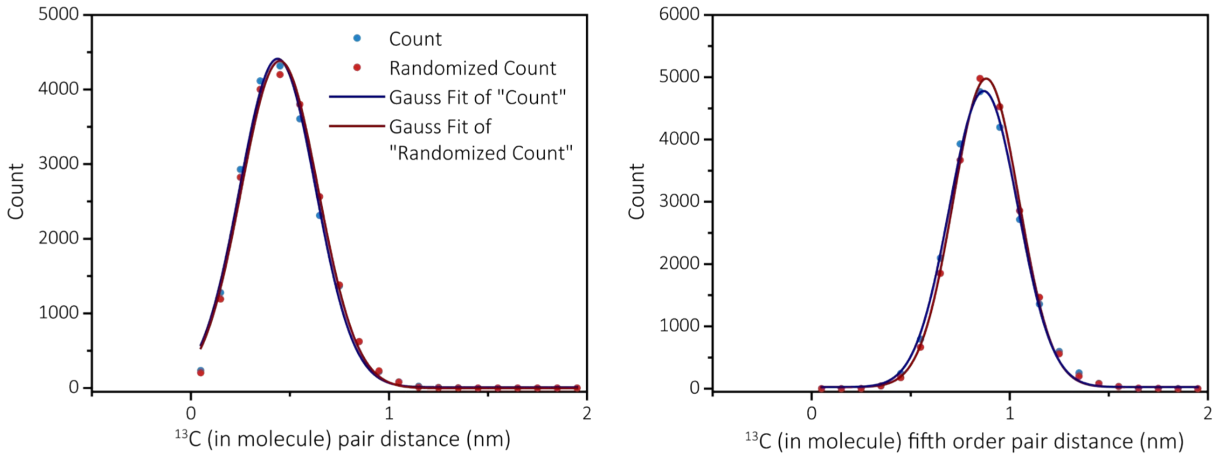

Figure 30: Fit of the randomized nearest neighbor distributions and measured nearest neighbor distributions of 15 min samples.

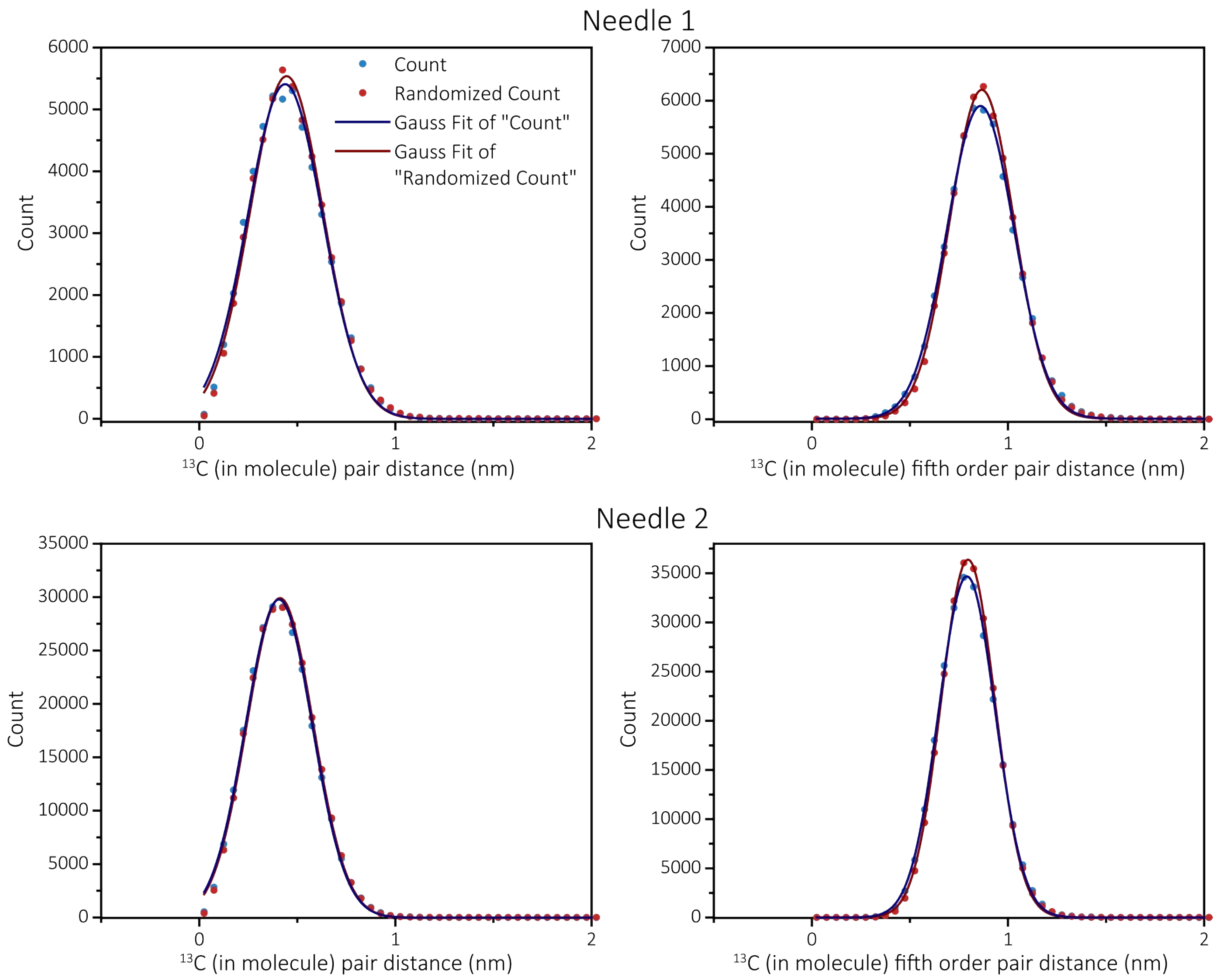

Figure S31: Fit of the randomized nearest neighbor distributions and measured nearest neighbor distributions of 30 min samples.

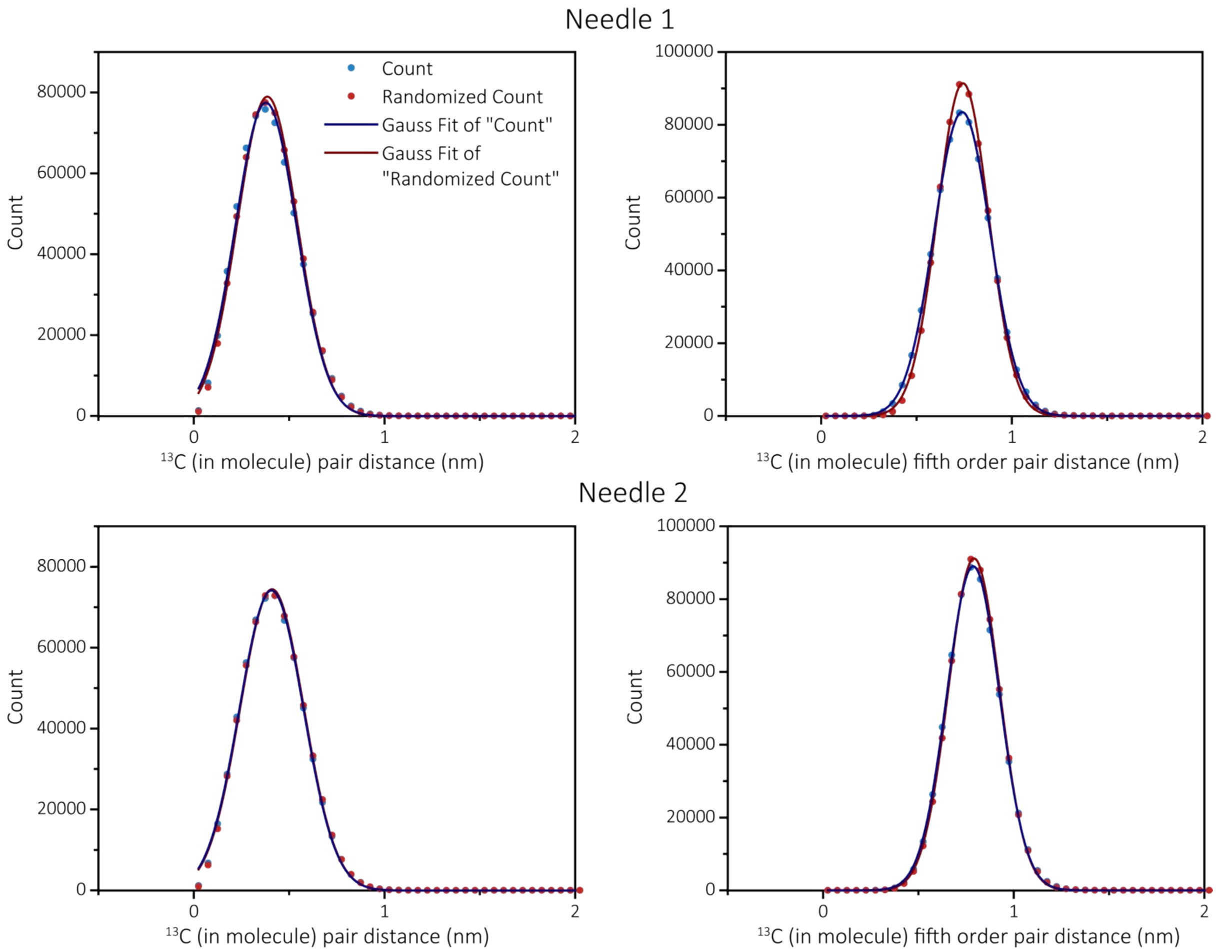

Figure S32: Fit of the randomized nearest neighbor distributions and measured nearest neighbor distributions of 60 min samples.

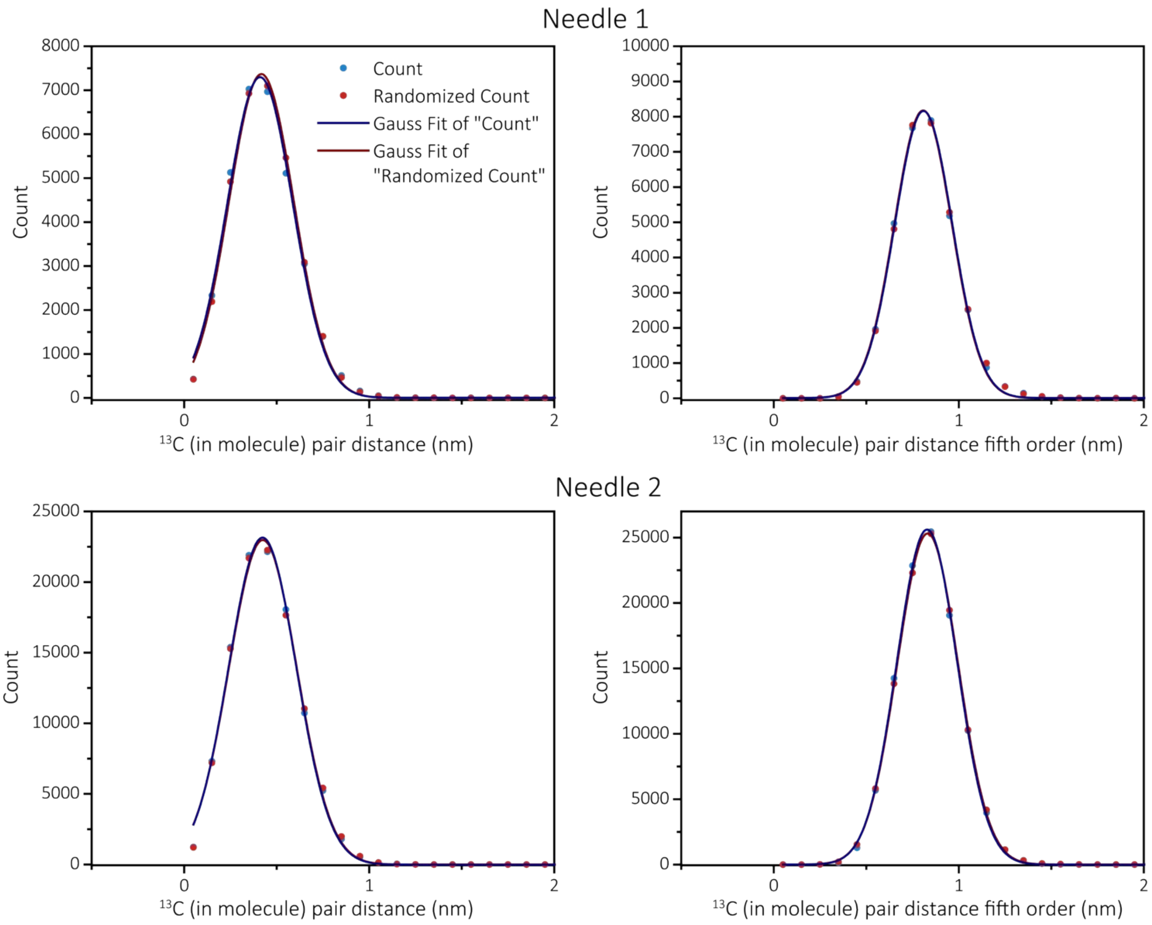

Figure S33: Fit of the randomized nearest neighbor distributions and measured nearest neighbor distributions of 120 min samples.

These fittings can help to test the Pearson coefficient as an effective method to quantify the deviation from random distribution. We were only able to do these fittings as the measured curves did not deviate that much from the random Gaussian curve. This is important as the measured distributions should be describable by a Gaussian curve as well.

The differences between the maxima of the Gaussian curves are compared to the Pearson coefficient changing with reaction time. There is a correlation between the two quantification methods.

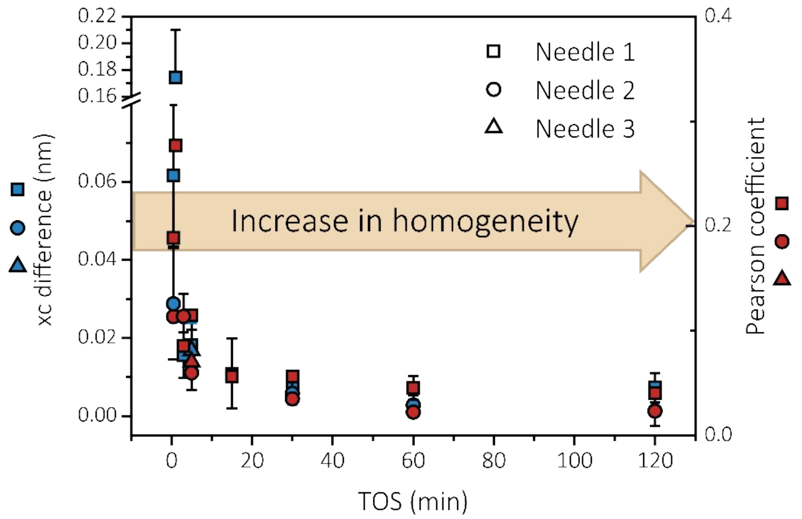

Figure S34: Pearson coefficient compared to the differences in maximum of the Gaussian curves. Error bars are calculated taking the quality of the fit into account.

S3.5 Radial distribution analysis

Another tool which was used to determine possible heterogeneities was the radial distribution function (RDF). This technique is especially suitable for determination of short distance affinities between elements.[1] In this technique, the local concentration is measured radially for a certain ion in the dataset. We have plotted the RDFs normalized to the bulk concentration within the APT dataset. Higher than bulk localized concentrations may indicate short length scale affinities.[1,6,7,8] In these figures, the elements represent the corresponding single atoms and molecules (decomposed) as explained in Section 4.1 (blue = Al single atoms and molecules, green = O single atoms and molecules, grey = Si single atoms and molecules and red = ^{13}C single atoms and molecules).

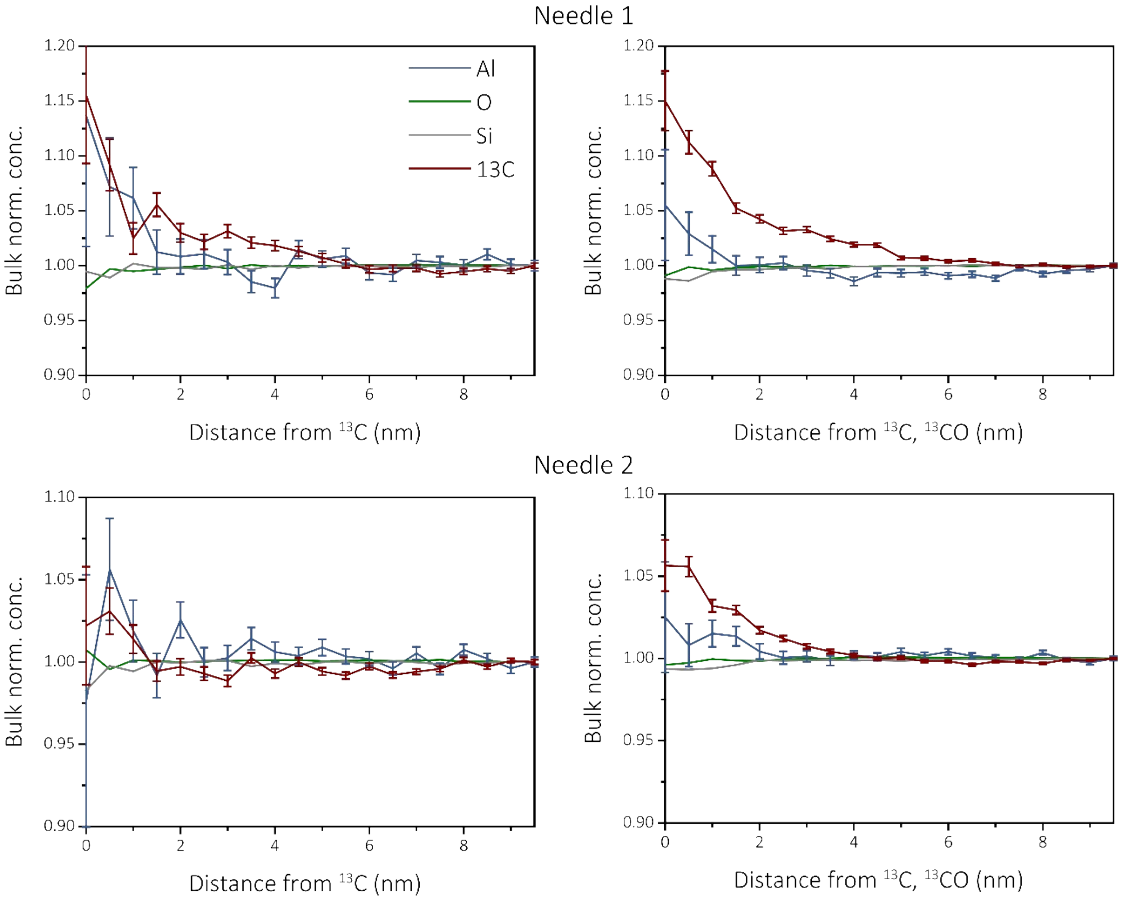

Figure S35: Radial distribution functions of H-SSZ-13 0.5 min coked sample needle 1 and 2 of ^{13}C and ^{13}CO atoms/molecules found in the MS.

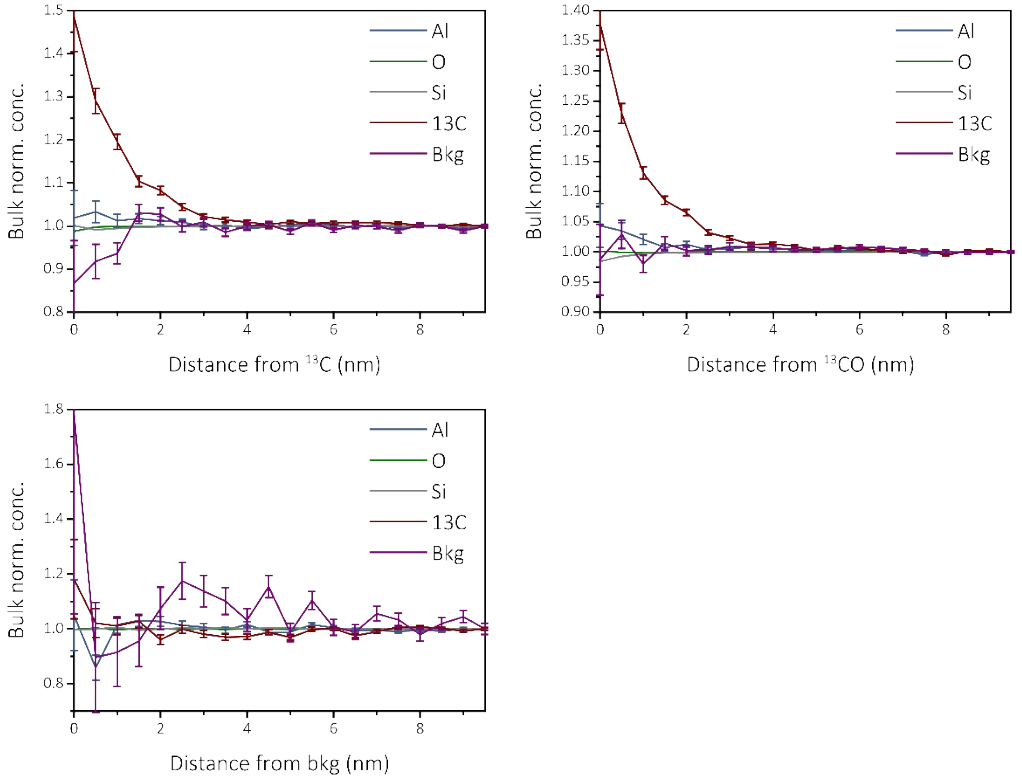

Figure S36: Radial distribution functions of H-SSZ-13 1 min coked sample needle 1 of ^{13}C and ^{13}CO and the background atoms/molecules found in the MS.

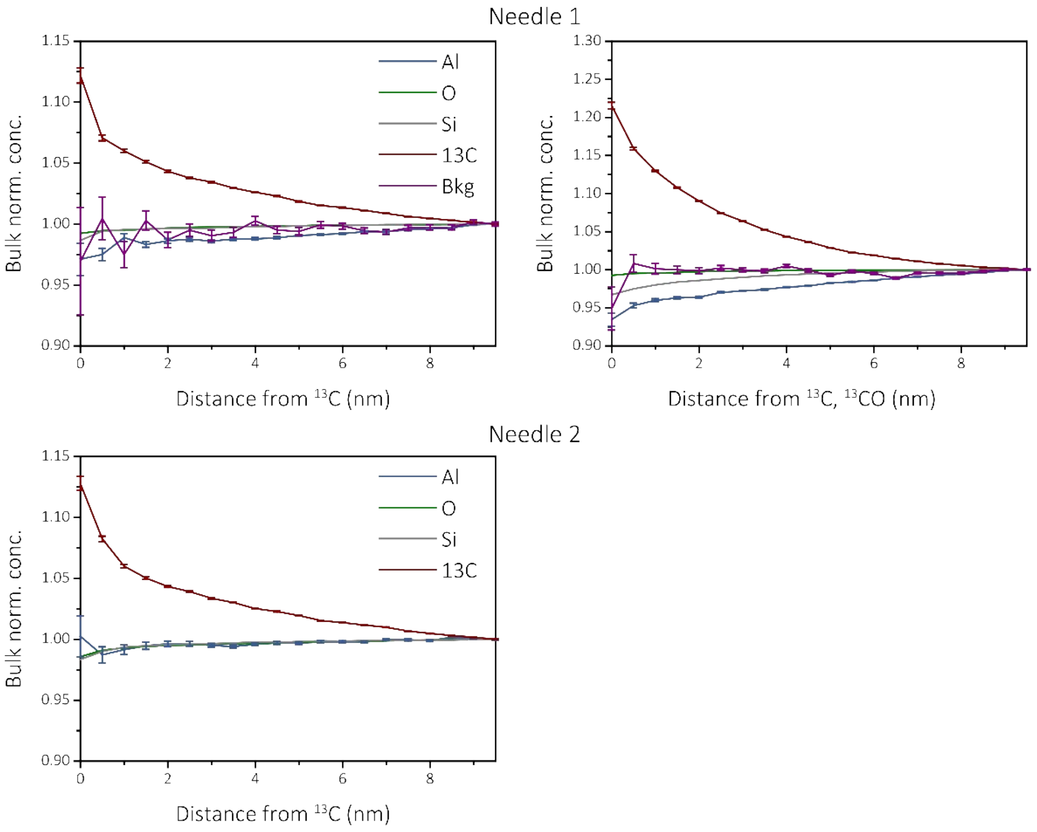

Figure S37: Radial distribution functions of H-SSZ-13 3 min coked sample needle 1 and 2 of ^{13}C and ^{13}CO atoms/molecules found in the MS.

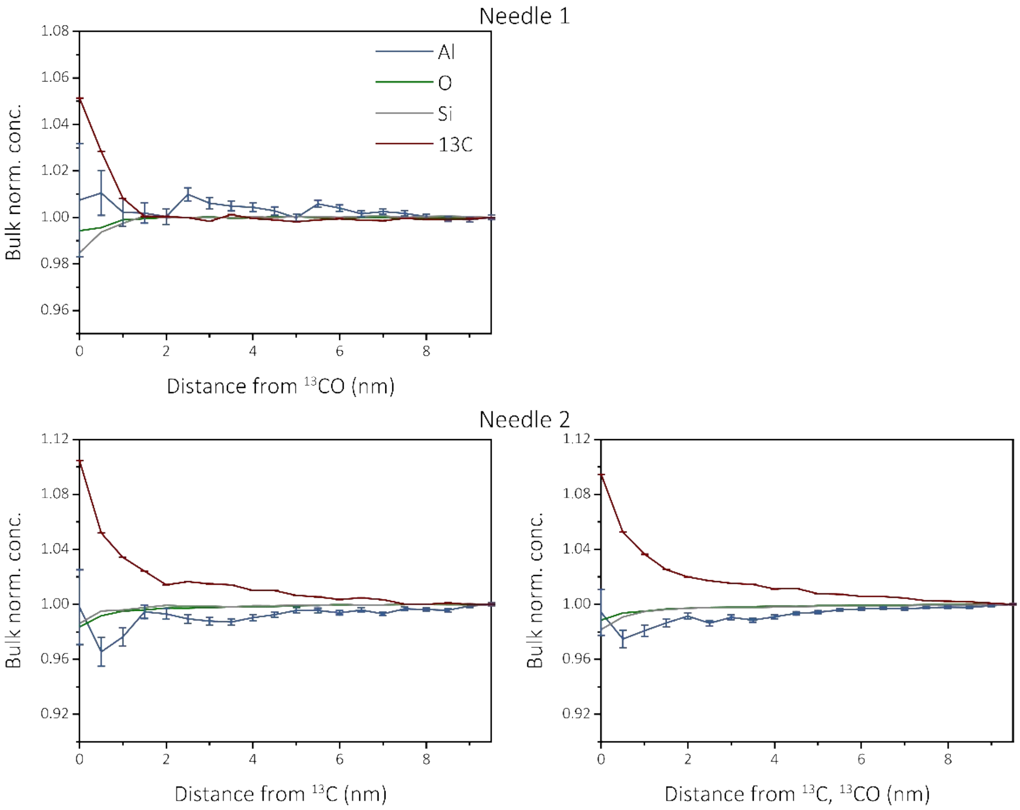

Figure S38: Radial distribution functions of H-SSZ-13 5 min coked sample needle 1 and 2 of ^{13}C and ^{13}CO atoms/molecules found in the MS.

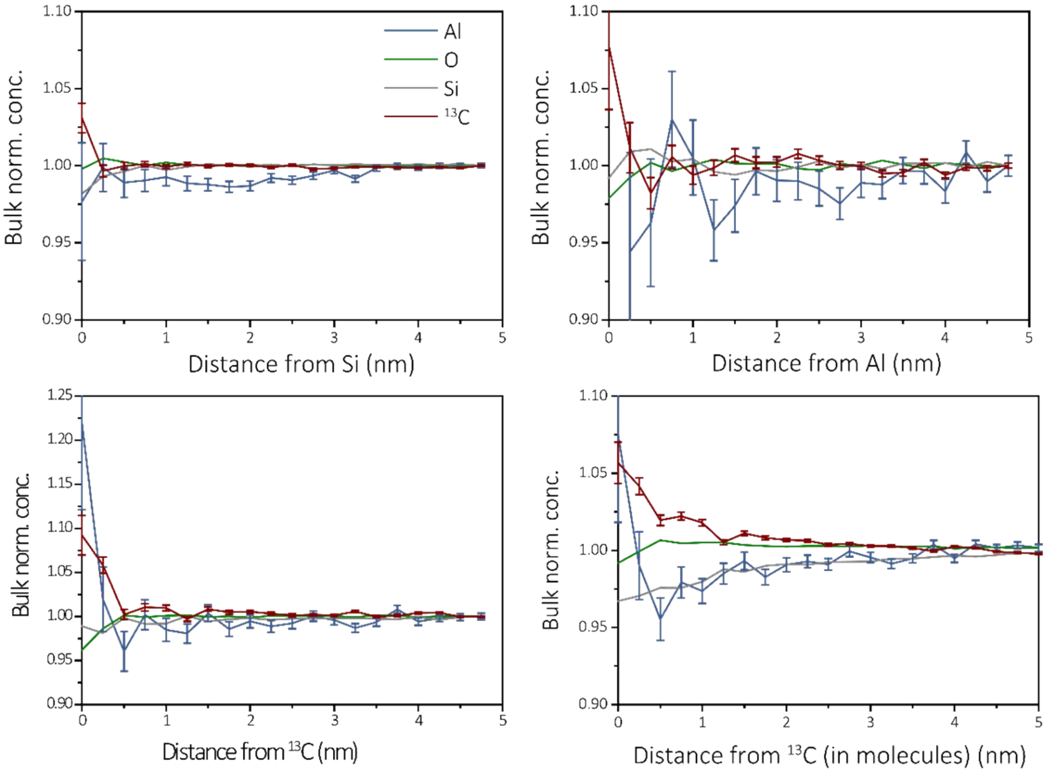

Figure S39: Radial distribution functions of H-SSZ-13 15 min coked sample needle 1 of Si, Al and ^{13}C decomposed from the molecules found in the MS and ^{13}C single atoms.

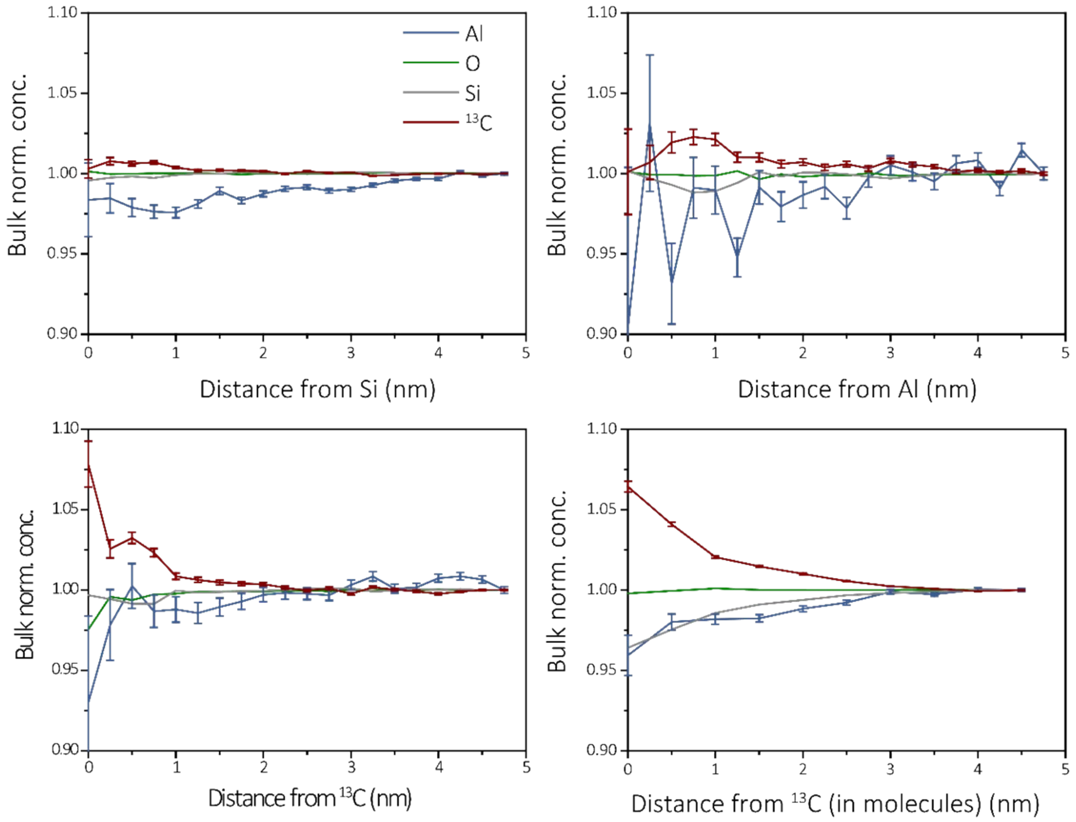

Figure S40: Radial distribution functions of H-SSZ-13 30 min coked sample needle 1 of Si, Al and ^{13}C decomposed from the molecules found in the MS and ^{13}C single atoms.

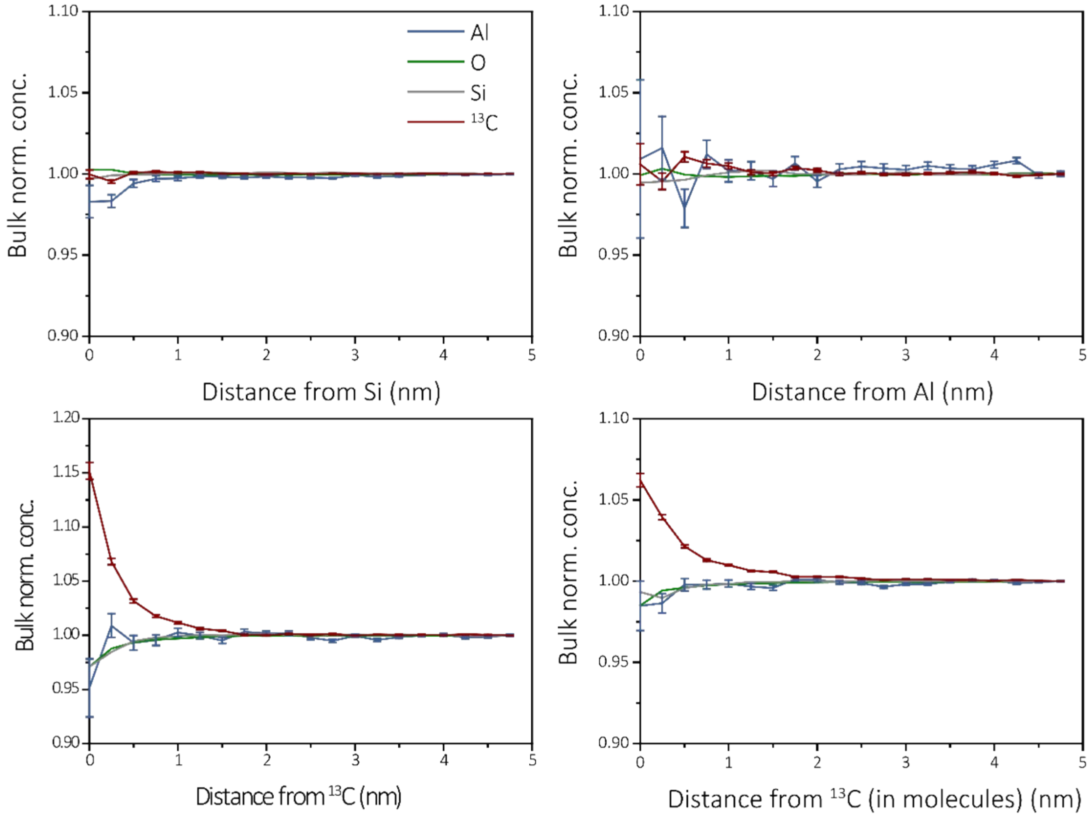

Figure S41: Radial distribution functions of H-SSZ-13 30 min coked sample needle 2 of Si, Al and ^{13}C decomposed from the molecules found in the MS and ^{13}C single atoms.

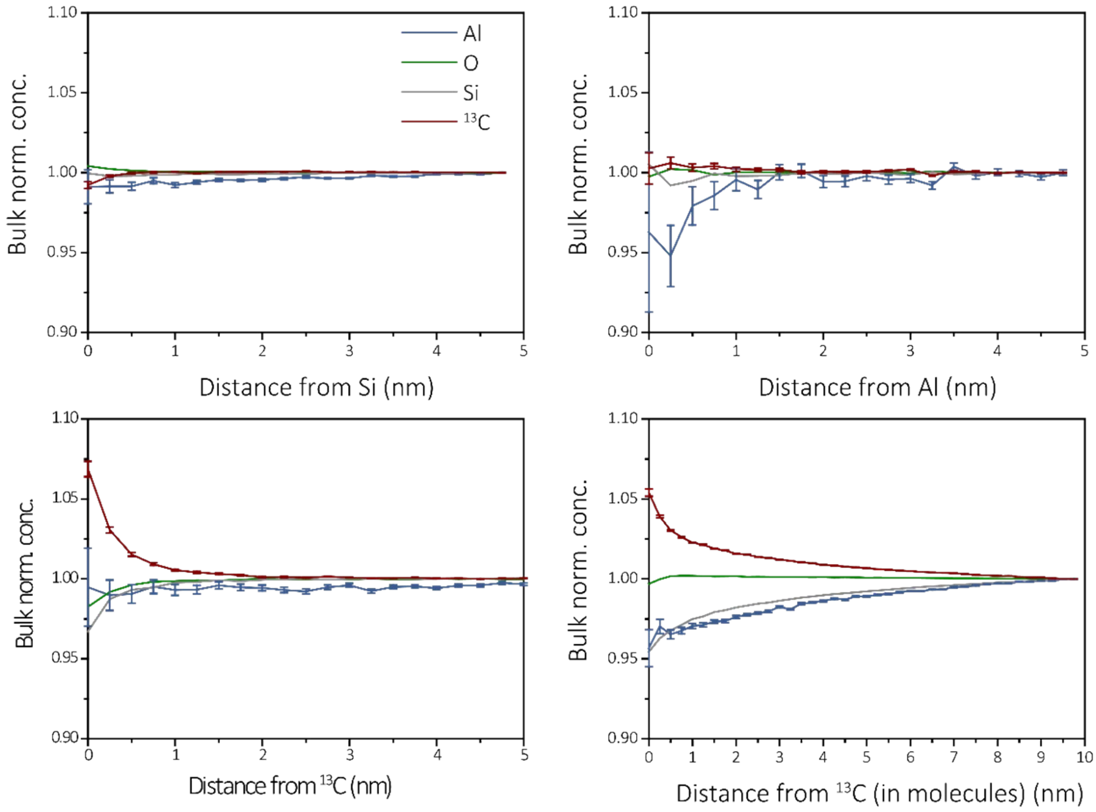

Figure S42: Radial distribution functions of H-SSZ-13 60 min coked sample needle 1 of Si, Al and ^{13}C decomposed from the molecules found in the MS and ^{13}C single atoms.

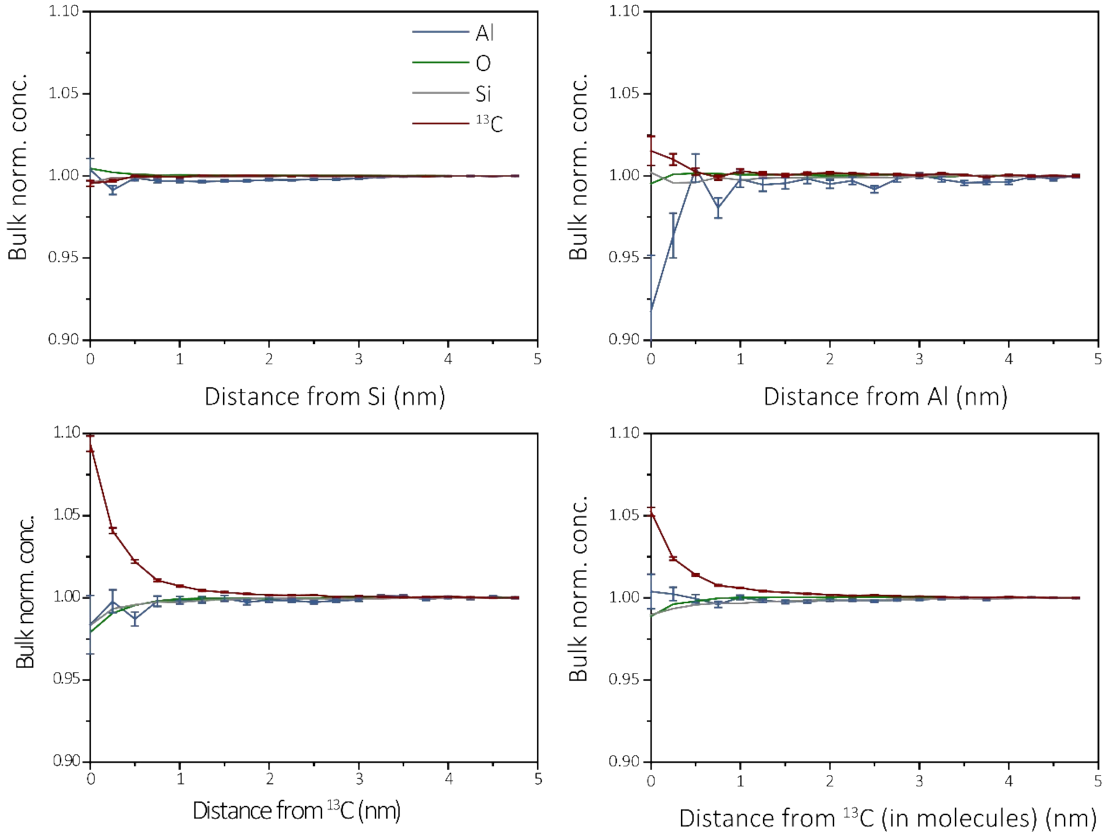

Figure S43: Radial distribution functions of H-SSZ-13 60 min coked sample needle 2 of Si, Al, ^{13}C single atoms and 13C decomposed molecules found in the MS.

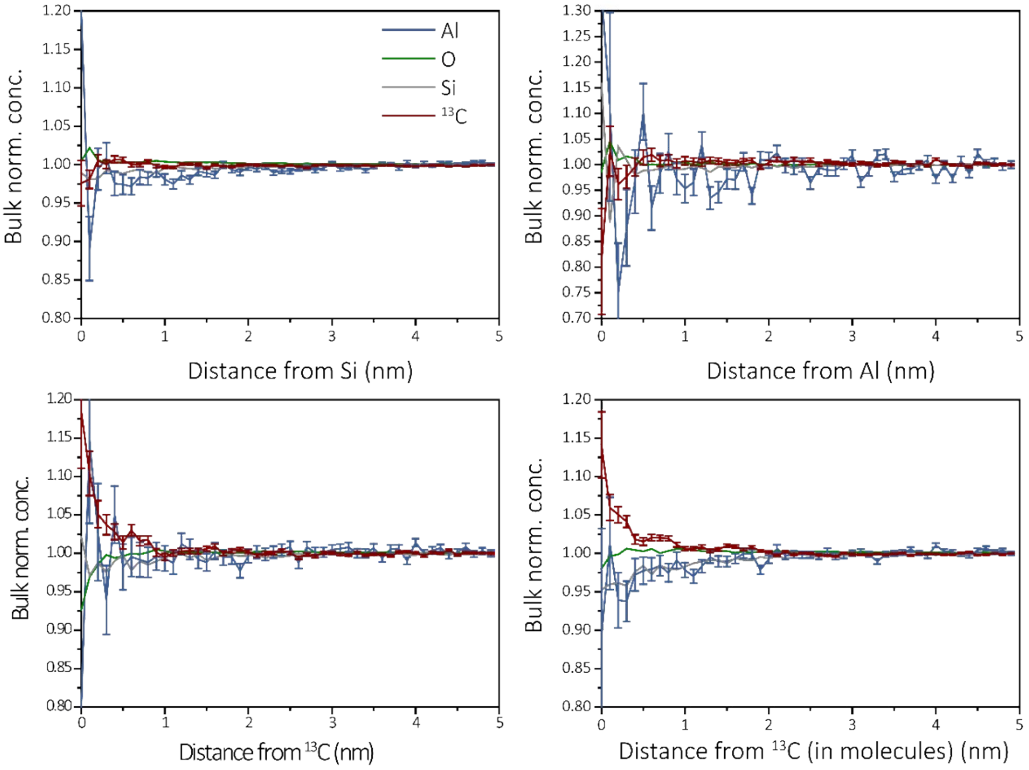

Figure S44: Radial distribution functions of H-SSZ-13 120 min coked sample needle 1 of Si, Al and ^{13}C decomposed from the molecules found in the MS and ^{13}C single atoms.

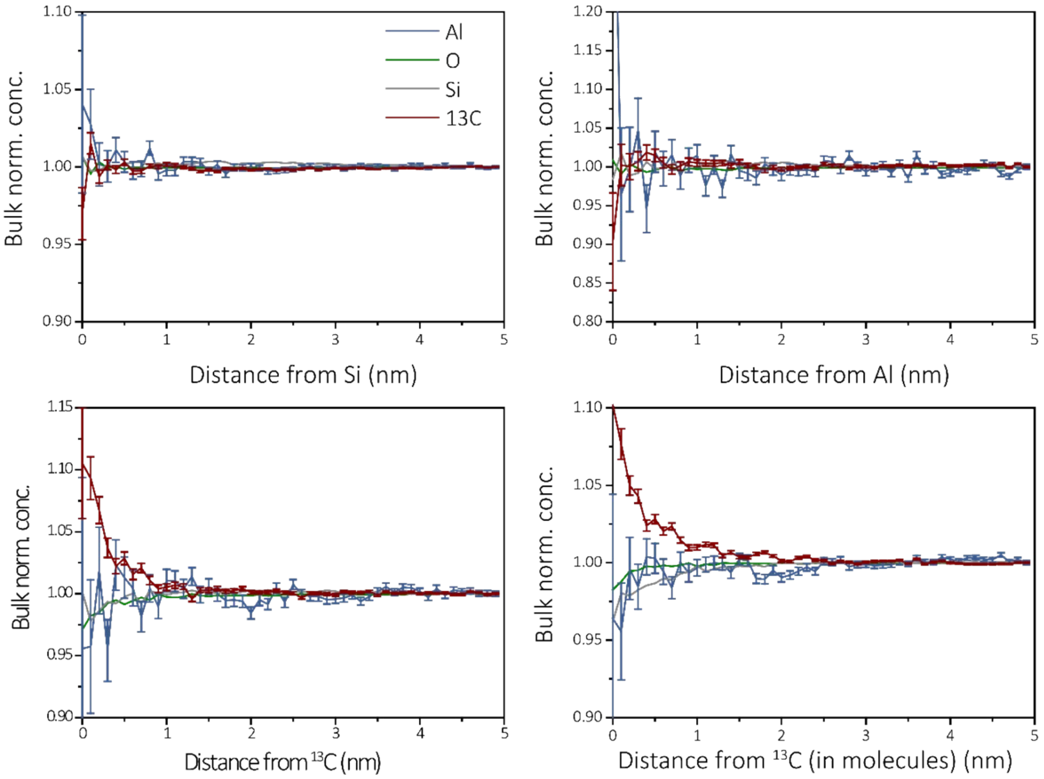

Figure S45: Radial distribution functions of H-SSZ-13 120 min coked sample needle 2 of Si, Al and ^{13}C decomposed from the molecules found in the MS and ^{13}C single atoms.

References

(1) Schmidt, J. E.; Peng, L.; Paioni, A. L.; Ehren, H. L.; Guo, W.; Mazumder, B.; Matthijs De Winter, D. A.; Attila, Ö.; Fu, D.; Chowdhury, A. D.; Houben, K.; Baldus, M.; Poplawsky, J. D.; Weckhuysen, B. M. Isolating Clusters of Light Elements in Molecular Sieves with Atom Probe Tomography. *J. Am. Chem. Soc.* **2018**, *140*, 9154–9158. https://doi.org/10.1021/jacs.8b04494.

(2) Tang, F.; Zhu, T.; Oehler, F.; Fu, W. Y.; Griffiths, J. T.; Massabuau, F. C. P.; Kappers, M. J.; Martin, T. L.; Bagot, P. A. J.; Moody, M. P.; Oliver, R. A. Indium Clustering in A-Plane InGaN Quantum Wells as Evidenced by Atom Probe Tomography. *Appl. Phys. Lett.* **2015**, *106*, 072104. https://doi.org/10.1063/1.4909514.

(3) Stephenson, L. T.; Moody, M. P.; Liddicoat, P. V.; Ringer, S. P. New Techniques for the Analysis of Fine-Scaled Clustering Phenomena within Atom Probe Tomography (APT) Data. *Microsc. Microanal.* **2007**, *13*, 448–463. https://doi.org/10.1017/S1431927607070900.

(4) Philippe, T.; De Geuser, F.; Duguay, S.; Lefebvre, W.; Cojocaru-Mirédin, O.; Da Costa, G.; Blavette, D. Clustering and Nearest Neighbour Distances in Atom-Probe Tomography. *Ultramicroscopy* **2009**, *109*, 1304–1309. https://doi.org/10.1016/j.ultramic.2009.06.007.

(5) Larson, D. J.; Prosa, T. J.; Ulfig, R. M.; Geiser, B. P.; Kelly, T. F. *Local Electrode Atom Probe Tomography*; Springer, New York, 2013.

(6) Booth-Morrison, C.; Mao, Z.; Diaz, M.; Dunand, D. C.; Wolverton, C.; Seidman, D. N. Role of Silicon in Accelerating the Nucleation of Al3(Sc,Zr) Precipitates in Dilute Al-Sc-Zr Alloys. *Acta Mater.* **2012**, *60*, 4740–4752. https://doi.org/10.1016/j.actamat.2012.05.036.

(7) Haley, D.; Petersen, T.; Barton, G.; Ringer, S. P. Influence of Field Evaporation on Radial Distribution Functions in Atom Probe Tomography. *Philos. Mag.* **2009**, *89*, 925–943. https://doi.org/10.1080/14786430902821610.

(8) Zhou, J.; Odqvist, J.; Thuvander, M.; Hedström, P. Quantitative Evaluation of Spinodal Decomposition in Fe-Cr by Atom Probe Tomography and Radial Distribution Function Analysis. *Microsc. Microanal.* **2008**, *19*, 666–675.
